# Structural and mechanistic insights into phospholipid transfer by Ups1–Mdm35 in mitochondria

**DOI:** 10.1038/ncomms8922

**Published:** 2015-08-03

**Authors:** Yasunori Watanabe, Yasushi Tamura, Shin Kawano, Toshiya Endo

**Affiliations:** 1 Faculty of Life Sciences, Kyoto Sangyo University, Kamigamo-motoyama, Kita-ku, Kyoto, 603-8555, Japan; 2 JST/CREST, Faculty of Life Sciences, Kyoto Sangyo University, Kamigamo-motoyama, Kita-ku, Kyoto, 603-8555, Japan; 3 JST/CREST, Research Center for Materials Science, Nagoya University, Chikusa-ku, Nagoya, 464-8602, Japan; 4 Research Center for Materials Science, Nagoya University, Chikusa-ku, Nagoya, 464-8602, Japan; 5 Department of Chemistry, Graduate School of Science, Nagoya University, Chikusa-ku, Nagoya, 464-8602, Japan

## Abstract

Eukaryotic cells are compartmentalized into membrane-bounded organelles whose functions rely on lipid trafficking to achieve membrane-specific compositions of lipids. Here we focused on the Ups1–Mdm35 system, which mediates phosphatidic acid (PA) transfer between the outer and inner mitochondrial membranes, and determined the X-ray structures of Mdm35 and Ups1–Mdm35 with and without PA. The Ups1–Mdm35 complex constitutes a single domain that has a deep pocket and flexible Ω-loop lid. Structure-based mutational analyses revealed that a basic residue at the pocket bottom and the Ω-loop lid are important for PA extraction from the membrane following Ups1 binding. Ups1 binding to the membrane is enhanced by the dissociation of Mdm35. We also show that basic residues around the pocket entrance are important for Ups1 binding to the membrane and PA extraction. These results provide a structural basis for understanding the mechanism of PA transfer between mitochondrial membranes.

## Abstract (web summary)

Phospholipid trafficking between membranes is essential to maintain the structural integrity and function of membrane-bound cellular compartments. Here the authors establish the structural basis for transport of phosphatidic acid between the outer and inner membranes of the mitochondria by the Ups1–Mdm35 lipid-transport complex.

## Graphical abstract

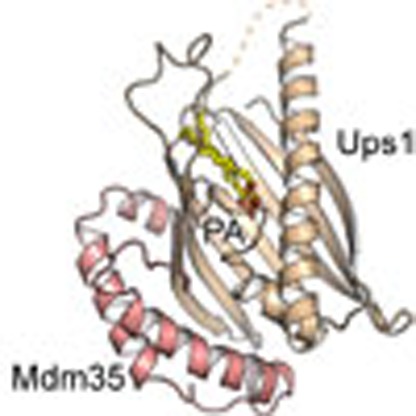

Eukaryotic cells are highly compartmentalized into membrane-bounded organelles with distinct functions, which strictly rely on the residence of organelle-specific proteins and lipid compositions. Phospholipids are mainly synthesized in the endoplasmic reticulum (ER) membrane and the inner membrane (IM) of mitochondria, and then distributed to all the cellular membranes to achieve specific lipid contents of each membrane^1^,^2^,^3^,^4^,^5^. How hydrophobic phospholipid molecules can traverse aqueous compartments to shuttle between different membranes, for example, the ER membrane and the mitochondrial IM via the mitochondrial outer membrane (OM) is a critical question concerning the mechanism of membrane biogenesis.

Mitochondria, a cellular powerhouse, require properly regulated abundance of phospholipids in the OM and IM, including a mitochondrial signature phospholipid cardiolipin, for their normal functions^6^,^7^. Cardiolipin is synthesized through several steps of conversion and modification of phosphatidic acid (PA) by a chain of enzymes localized in the mitochondrial IM. PA is first converted to the high-energy intermediate phospholipid, CDP–diacylglycerol (CDP–DAG) by mitochondrial CDP–DAG synthase Tam41 (ref. 8), and then Pgs1 generates phosphatidylglycerol phosphate (PGP) from CDP–DAG and glycerol 3-phosphate^9^. Gep4 dephosphorylates PGP to produce phosphatidylglycerol (PG), which is coupled with CDP–DAG to form cardiolipin by cardiolipin synthase Crd1 (refs 10, 11, 12). Cardiolipin is further remodelled by deacylation and reacylation that generate mature cardiolipin with unsaturated fatty acids^13^,^14^,^15^,^16^. Importantly, PA is synthesized on the ER membrane because PA synthetic enzymes such as lyso-PA acyltransferases Slc1 and Ale1 and a DAG kinase Dgk1 are mainly localized in the ER^17^,^18^,^19^. Therefore, PA needs to be transported from the ER membrane to the mitochondrial IM by crossing the mitochondrial OM for cardiolipin biosynthesis.

Recently, Ups1, a yeast member of the conserved mitochondrial intermembrane space (IMS) proteins, was shown to mediate PA transfer between the mitochondrial OM and IM in cooperation with Mdm35 (refs 20, 21). Ups1 was originally identified as a protein responsible for membrane topogenesis of Mgm1, a mitochondrial fusion protein in the IM^22^. Loss of Ups1 was then found to lead to decrease in the cardiolipin level, yet simultaneous deletion of Ups1 and its homolog Ups2 restored the decreased cardiolipin level, suggesting antagonistic functions of Ups1 and Ups2 (refs 23, 24, 25). Ups2 was also found to be important for maintaining normal PE levels independently of Ups1 (refs 23, 24, 25). Mdm35, another conserved yeast IMS protein, is a binding partner of Ups proteins, and facilitate their import into mitochondria^26^,^27^. In addition to the role in the Ups protein import, Mdm35 association with Ups1 even after its import appears to be important for regulation of the Ups1 function for PA transfer^20^. However, how Ups1 and Mdm35 cooperate to recognize PA and drive transport of PA from the OM to IM by traversing the aqueous IMS remains unclear.

To gain mechanistic insights into how Ups1 with Mdm35 exerts its PA transfer activity, we determined the X-ray crystal structures of Mdm35 and the Ups1–Mdm35 complex with and without PA. Surprisingly, two distinct polypeptides of Ups1 and Mdm35 form a single compact domain, the Ups1 part of which corresponds to the START (StAR-related lipid-transfer) domain^28^,^29^, likely functioning as a lipid-binding domain. Like other START domain-containing proteins, the Ups1–Mdm35 complex contains a deep pocket that can accommodate a lipid molecule and an Ω-loop likely functioning as a lid for the pocket^30^,^31^,^32^. The structure of the Ups1–Mdm35 complex with PA shows that the PA molecule binds to the pocket in a head-in manner to interact with positively charged residues in the pocket. Parallel liposome binding and lipid transfer assays by using Ups1 mutants with Mdm35 show that Arg residue at the bottom of the pocket and the Ω-loop lid are critical for the PA transfer activity and that conserved Lys residues around the entrance of the pocket are important for binding to cardiolipin-containing liposome and PA transfer. The crystal structures of Ups1–Mdm35 with and without PA provide a basis for understanding the molecular mechanism of phospholipid transfer within mitochondria.

## Results

### Structure of Mdm35

**Figure 1:**
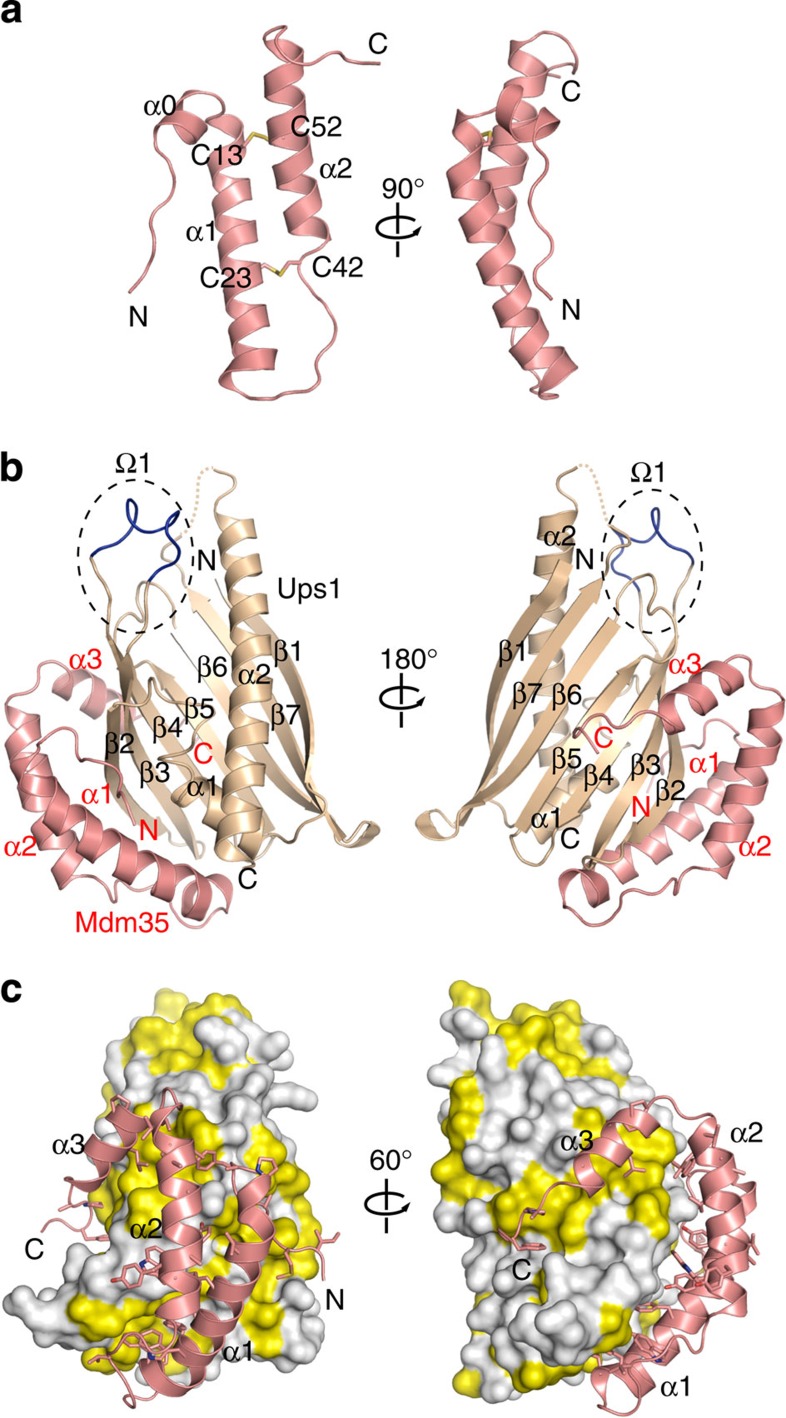
Crystal structures of Mdm35 and the Ups1–Mdm35 complex. (**a**) A ribbon diagram of the structure of Mdm35. The four Cys residues and α-helices are labelled. (**b**) A ribbon diagram of the structure of the Ups1–Mdm35 complex. Ups1 and Mdm35 are coloured in light brown and pink, respectively, and Ω-loop of Ups1 in blue. The α-helices, β-strands, and Ω-loop are labelled. (**c**) The Ups1 (molecular surface)–Mdm35 (ribbon diagram) complex viewed from different angles. Hydrophobic residues of Ups1 are coloured in yellow, and those of Mdm35 are shown by stick model with N blue and O red.

Previous studies showed that binding of Mdm35 to Ups1 facilitates translocation of Ups1 across the OM and regulates PA transfer between the OM and IM. To understand the mechanism underlining these Mdm35 functions, we attempted to determine the crystal structure of *Saccharomyces cerevisiae* Mdm35. Although full-length Mdm35 (86 residues) failed to crystallize, Mdm35ΔC5 lacking the C-terminal five residues grew crystals suitable for structural analyses. The crystal structure of Mdm35ΔC5 was thus determined from its seleno-methionine (SeMet)-labelled protein crystal by the multiwavelength anomalous dispersion method using the anomalous signal of selenium atoms and was subsequently refined to 1.45-Å resolution against the data set from the native crystal (Fig. 1a, Table 1). Residues 67–81 did not exhibit defined electron density, suggesting that the C-terminal segment is disordered in solution. The crystal structure of Mdm35ΔC5 is hereafter referred to as the Mdm35 structure. Mdm35 possesses a twin CX_9_C motif, which is indicative of the substrate for the Tim40/MIA import pathway^33^. Mdm35 contains a short α-helix (α0) and two long α-helices (α1 and α2), which form an antiparallel α-hairpin that is covalently paired by two disulfide bridges, Cys13–Cys52 and Cys23–Cys42. The antiparallel α-hairpin fold is typically found in twin CX_9_C motif containing proteins such as Tim40 and Cox17 (ref. 33).

### Structure of the Ups1–Mdm35 complex

We next attempted to determine the structure of *S. cerevisiae* Ups1 in a complex with Mdm35. Ups1 can be stably expressed in *Escherichia coli* cells only with coexpression of Mdm35 (ref. 20). The full-length Ups1 (175 amino acids) and Mdm35 failed to cocrystallize, yet Ups1ΔC5 lacking the C-terminal five residues and Mdm35ΔC5 grew cocrystal. We hereafter call the Ups1ΔC5–Mdm35ΔC5 complex as the Ups1–Mdm35 complex. The crystal structure of the Ups1–Mdm35 complex was determined from its SeMet-labelled protein crystal by the single-wavelength anomalous dispersion method using the anomalous signal of selenium atoms, and was subsequently refined to 1.40-Å resolution against the data set from the native crystal (Fig. 1b, Table 1). The asymmetric unit of the crystal contains two Ups1–Mdm35 heterodimers, which have a similar conformation to each other, with root mean square (r.m.s.) differences of 0.6 and 0.3 Å for 168 and 72 Cα atoms of Ups1 and Mdm35, respectively. In the asymmetric unit, two Ups1 molecules were found to form a domain-swapped dimer (Supplementary Fig. 1a); the two Ups1 molecules exchange the identical C-terminal helix (residues 135–170) each other. However, gel-filtration analysis showed that the apparent MW of the Ups1–Mdm35 complex is about 30 kDa in solution, which is close to the MW of a monomeric Ups1–Mdm35 dimer (31 kDa). Besides, Cys residues introduced at positions 159 and 28, which are located in the exchanged C-terminal segment and the remaining part of the domain-swapped dimer, respectively, formed an intramolecular, not intermolecular, disulfide bond (Supplementary Fig. 1b,c). Therefore, the domain-swapped conformation likely arose from the crystallization artifact, and we thus refer to the monomeric Ups1–Mdm35 heterodimer, in which the C-terminal segment is connected to the polypeptide of the remaining part in the same molecule, as the Ups1–Mdm35 complex.

Ups1 contains two α-helices (α1 and α2) and a seven-stranded antiparallel β-sheet, which is folded into a half-barrel structure (β1-β7) and an Ω-loop (Ω1, residues 62–71) is formed between the β3 and β4 strands (Fig. 1b). Like free Mdm35, Mdm35 in the complex with Ups1, takes an antiparallel α-hairpin (α1 and α2), yet the C-terminal segment, which is disordered in free Mdm35, forms an α-helix (α3). Mdm35 embraces Ups1 with the three α helices, covering ∼1,400 Å^2^ (12% of the total surface area of the Ups1 molecule), rendering the Ups1–Mdm35 complex folded into a single-domain like structure (Fig. 1c).

### Ups1–Mdm35 interactions

**Figure 2:**
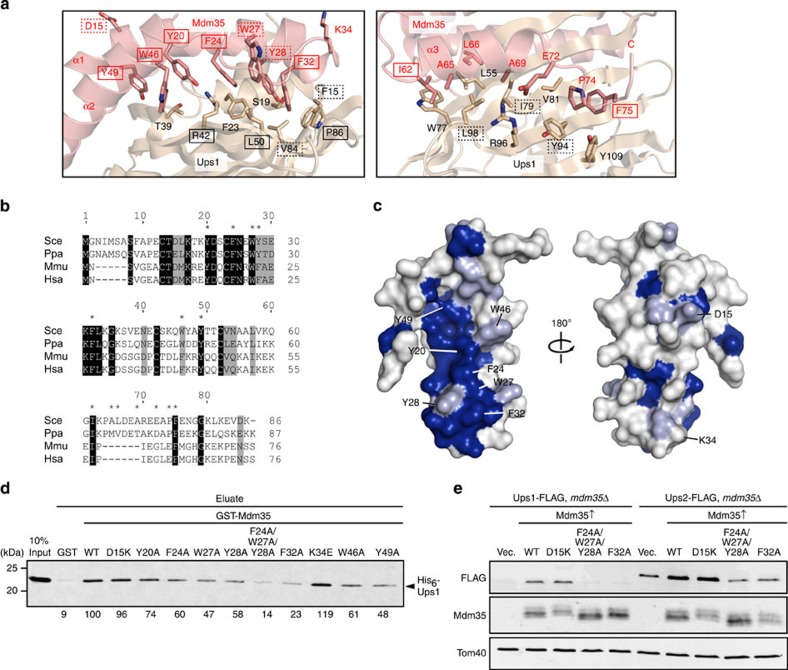
Interactions of Ups1 and Mdm35. (**a**) Interaction surface between Ups1 and Mdm35. Residues in Ups1 and Mdm35 are labelled in black and red, respectively. Conserved residues and type-conserved residues are indicated by boxes and dashed boxes, respectively. (**b**) Sequence alignment of Mdm35 homologues. Gaps are introduced to maximize the similarity. Conserved residues are shaded in black, and Type-conserved residues in grey. Residues marked by asterisks are involved in the interaction with Ups1. *Sce*, *Saccharomyces cerevisiae*; *Ppa*, *Pichia pastoris*; *Mmu*, *Mus musculus*; *Hsa*, *Homo sapiens.* (**c**) Mapping of the residues conserved (blue) or type-conserved (light blue) among the Mdm35 homologues on the Mdm35 structure. (**d**) 10 μM of GST-Mdm35 mutants and 10 μM of His_6_–Ups1–Mdm35 in 20 mM Tris-HCl, pH 7.5, and 150 mM NaCl were incubated with 50 μl of GS4B resin at 37 °C for 15 min. Proteins bound to the resin were eluted by 10 mM glutathione and analysed by SDS-PAGE followed by immunoblotting with the anti-His-tag antibody. Amounts of eluted proteins were quantified and shown below the gel (WT is set to 100). (**e**) *mdm35*Δ cells expressing Ups1-FLAG or Ups2-FLAG and indicated Mdm35 mutants (under the control of the *GAL1* promoter) were grown in SCGal containing 2% sucrose. Whole-cell lysates were subjected to SDS–PAGE and immunoblotting with the indicated antibodies.

A closer look at the crystal structure of the Ups1–Mdm35 complex indicates that the hydrophobic side chains of Tyr20, Phe24, Trp27, Tyr28 and Phe32 in the α1 helix and Trp46 and Tyr49 in the α2 helix of Mdm35 bind to the hydrophobic region formed by Phe15, Ser19, Phe23, Thr39, Arg42, Leu50, Val84 and Pro86 of Ups1 (Fig. 2a, left panel). The induced C-terminal α-helix α3 of Mdm35 binds to the region formed by the β4, β5 and β6 strands of Ups1 (Fig. 2a, right panel). The hydrophobic Ups1-binding residues in the α1 and α2 helices of Mdm35 are well-conserved (Fig. 2b, asterisk) and aligned to form a concave surface of Mdm35 (Fig. 2c), which fits the complementary convex curvature of the Ups1 surface (Fig. 1c). The hydrophobic Mdm35-binding residues of Ups1 are also well conserved (Fig. 2a).

We then tested the effects of mutations in the conserved hydrophobic regions of Mdm35 on the Ups1–Mdm35 interactions *in vitro*. The full-length Ups1–Mdm35 complex was incubated with the glutathione *S*-transferase (GST)–Mdm35 fusion proteins with various mutations and subjected to affinity purification by Glutathion Sepharose affinity chromatography for the GST tag (Fig. 2d). Copurified Ups1 reflects the exchange of Mdm35, originally bound to Ups1, with mutant GST-Mdm35. Ala mutations of the conserved hydrophobic residues of Mdm35, especially the F24A/W27A/Y28A and F32A mutations, were thus found to abrogate interactions between Ups1 and Mdm35. In contrast, reverse-charge mutations of the charged residues on the opposite side of the α-helices did not impair the Ups1–Mdm35 interactions. Since Mdm35 functions as a binding trap in the IMS to facilitate import of Ups proteins into the IMS and non-imported Ups proteins are not stable^26^, we also tested the effects of these Mdm35 mutations on the protein levels of Ups1 and Ups2 *in vivo*. The F24A/W27A/Y28A and F32A mutations, but not the D15K mutation, significantly decreased the levels of Ups1 and Ups2, indicating decreased binding abilities of Mdm35 for Ups1 and Ups2 (Fig. 2e). Sequence alignment between Ups1 and Ups2 showed that the Ups1 residues indicated for interactions with Mdm35 are moderately conserved in Ups2 (Supplementary Fig. 2). These results suggest that Ups2 interacts with Mdm35 in a similar manner to Ups1.

### Positively charged pocket of Ups1

**Figure 3:**
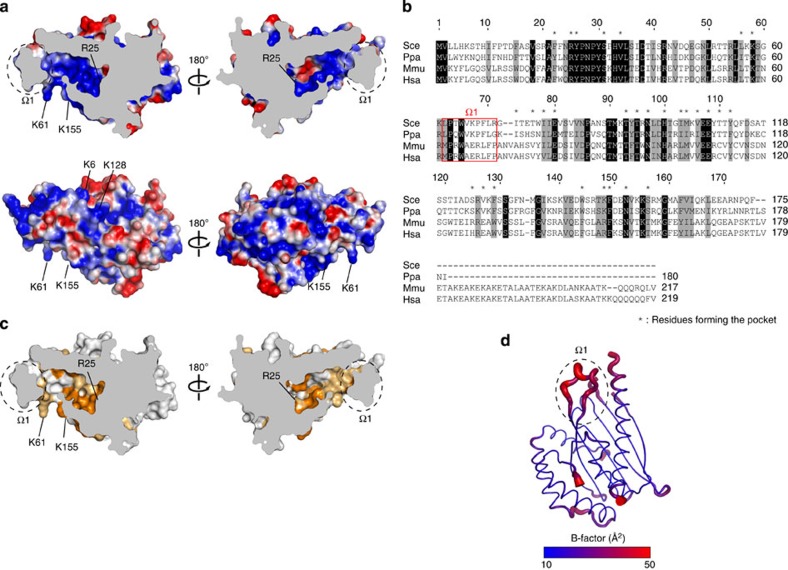
Lid and pocket of Ups1–Mdm35. (**a**) Cutaway representation of the Ups1–Mdm35 complex at the level of the pocket, and coloured according to the surface electrostatic potential. (**b**) Sequence alignment of Ups1 homologues. The conserved residues and type-conserved residues are shaded in black and grey, respectively. Residues constituting the Ω1 loop are indicated by red box, and residues forming the pocket by asterisks. *Sce*, *S. cerevisiae*; *Ppa*, *P. pastoris*; *Mmu*, *M. musculus*; *Hsa*, *H. sapiens.* (**c**) Mapping of the residues conserved among the Ups1 homologues (orange, conserved residues; light orange, type-conserved residues) on the molecular surface of Ups1–Mdm35 in a cutaway representation as in **a**. (**d**) The crystallographic B-factors of Ups1–Mdm35 are coloured in a gradient ranging from blue (10 Å^2^) to red (50 Å^2^).

Structural comparison by the Dali search engine^34^ indicates that Ups1 complexed with Mdm35 is structurally similar to START domains in several lipid-transfer proteins, including a ceramide transport protein CERT^32^ and a phosphatidylinositol transfer protein PITPα^30^,^31^. This is consistent with the suggested role of Ups1 in PA transfer within mitochondria. Indeed like other START domains, Ups1 contains a positively charged, deep pocket with the dimension of 8 × 10 × 25 Å (∼2,000 Å^3^), to which lipid molecules could bind (Fig. 3a). The pocket is lined by 14 hydrophobic residues and 15 polar and/or charged residues (Fig. 3b, asterisk), most of which are evolutionally conserved (Fig. 3c orange and light-orange residues). In particular, Arg25 at the bottom of the pocket and Lys61 and Lys155 near the entrance of the pocket are well conserved. The entrance of the pocket is nearly closed by the Ω1 loop. The crystallographic B-factors of loop Ω1 are high as compared with those for the other regions, suggesting its flexible nature (Fig. 3d). As having been speculated for other START domains with such a flexible Ω loop, the loop Ω1 of Ups1 may function as a lid that regulates lipid binding to the pocket (see below).

### Structure of Ups1–Mdm35 with PA

**Figure 4:**
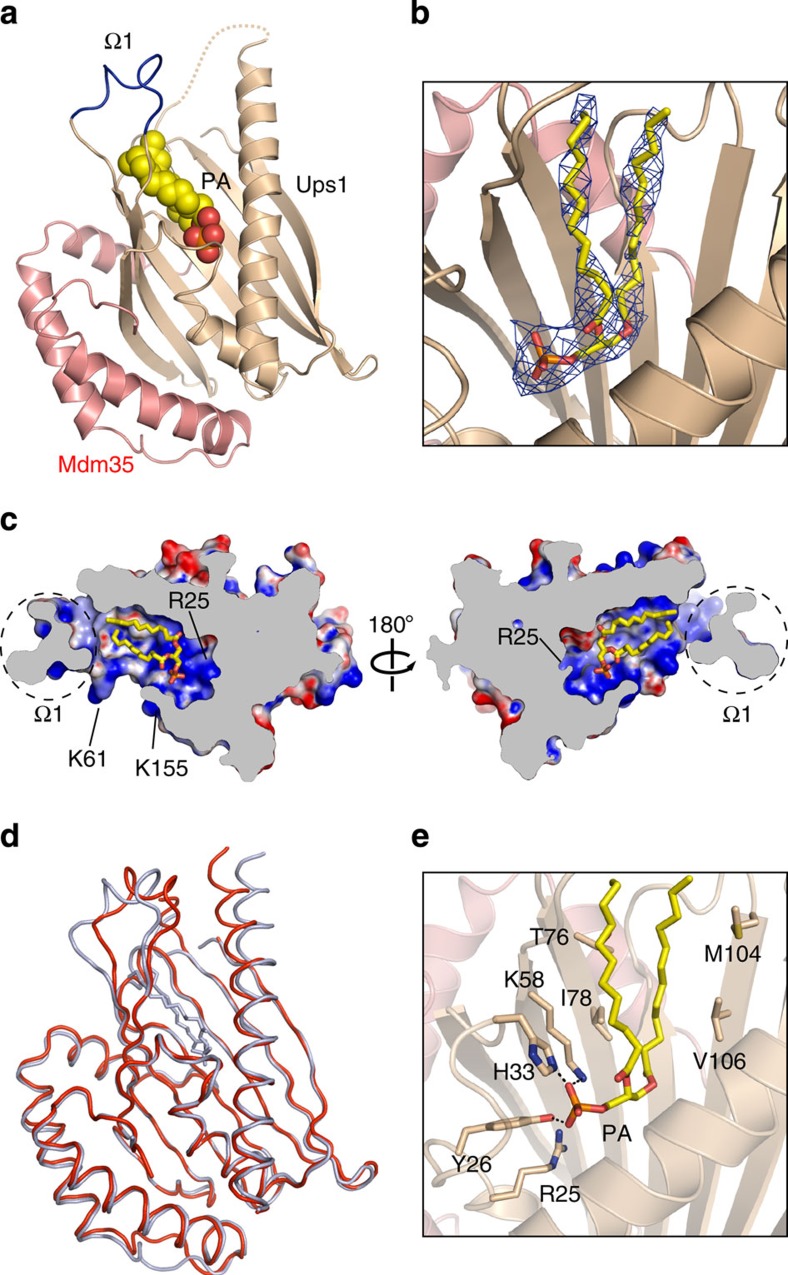
Structure of the Ups1–Mdm35–PA complex. (**a**) Ribbon diagram of the Ups1–Mdm35 complex with DLPA. The DLPA molecule is shown in space-filling form with C yellow, O red and P orange. (**b**) Electron density map of the DLPA molecule. The simulated annealing *F*_o_–*F*_c_ difference Fourier map was calculated by omitting the DLPA molecule, and is shown with blue meshes countered at 3.0σ. DLPA is superimposed in stick model. (**c**) Bound DLPA in stick model with the cutaway representation of Ups1–Mdm35 as in Fig. 3a. (**d**) Superposition of the apo-form (red) and PA-bound form (light blue) of Ups1–Mdm35. (**e**) Magnified view showing the detailed interaction around the phosphate group of the DLPA molecule. Residues responsible for DLPA recognition are shown as stick models. Broken lines designate possible hydrogen bonds.

To further investigate the role of the pocket of Ups1 in PA transfer, we attempted to co-crystallize the Ups1–Mdm35 complex with PA with different acyl chains, including POPA (1-palmitoyl-2-oleoyl-*sn*-glycero-3-phosphate) (16:0–18:1), DPPA (1,2-dipalmitoyl-sn-glycero-3-phosphate) (16:0–16:0), DMPA (1,2-dimyristoyl-*sn*-glycero-3-phosphate) (14:0–14:0), DLPA (1,2-dilauroyl-*sn*-glycero-3-phosphate) (12:0–12:0), DDPA (1,2-didecanoyl-*sn*-glycero-3-phosphate) (10:0–10:0) and DHPA (1,2-dihexanoyl-sn-glycero-3-phosphate) (6:0–6:0). Among these PA species, we could obtain crystals of the Ups1–Mdm35 complex with POPA and DLPA. Although we could not obtain good diffraction data of the Ups1–Mdm35 complex with POPA suitable for high-resolution structure determination, we successfully determined the crystal structure of the Ups1–Mdm35 complex with DLPA at 3.20 Å resolution by the molecular replacement method with the Ups1–Mdm35 apo-complex as a search model. The crystallographic asymmetric unit contained eight Ups1–Mdm35 molecules, and two of the eight Ups1–Mdm35 molecules contained a DLPA molecule in the pocket (Fig. 4a–c) while clear electron density was not observed in the pocket of the remaining six Ups1–Mdm35 molecules. The DLPA bound to the Ups1–Mdm35 molecule with the most defined electron density is subjected to further structural analysis and hereafter referred to as the bound DLPA. Structural comparison of the Ups1–Mdm35 complexes with and without a bound PA molecule shows the r.m.s. differences of 1.2 and 0.6 Å for 169 Cα atoms of Ups1 and 73 Cα atoms of Mdm35, respectively, indicating that no significant conformational change occurs in Ups1–Mdm35 on PA binding (Fig. 4d). Interestingly, some of the Ups1–Mdm35 molecules without clear electron density of DLPA in the crystallographic asymmetric unit had the Ω1 lid swung away from the pocket (Supplementary Fig. 3).

The bound DLPA molecule is completely enclosed in the pocket of Ups1, with the phosphate head positioned at the bottom of the pocket and the acyl-chain tails oriented toward the entrance of the pocket, yet leaving some empty space in the pocket (Fig. 4c). At the bottom of the pocket, the positively charged side chains of Arg25 and Lys58 are close to the negatively charged phosphate group of DLPA, and side chains of Tyr26, His33 and Lys58 are within the distance that allows formation of hydrogen bonds with the phosphate oxygen atoms (Fig. 4e). The hydrophobic acyl-chain tails are in contact with hydrophobic side chains of Thr76, Ile78, Met104 and Val106 in the pocket (Fig. 4e).

### Lid and basic residues are important for PA transfer

**Figure 5:**
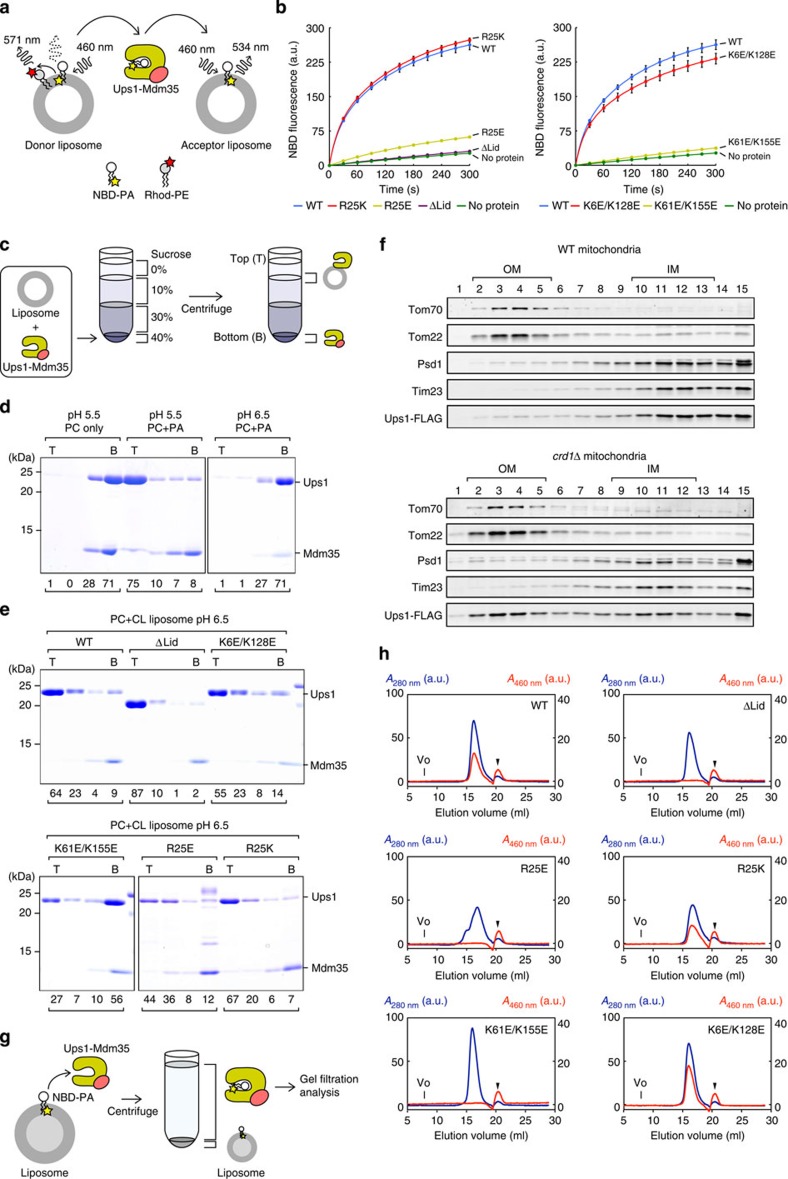
Lipid binding, PA extraction, and PA transfer activities of Ups1 mutants. (**a**) A schematic diagram of the fluorescent-based PA transfer assay between liposomes (see details in Methods). (**b**) PA transfer activities of the Ups1–Mdm35 complex for wild-type (WT) and the indicated mutant Ups1 were measured at 25 °C by the assay shown in **a**. At 0 s, the protein or buffer was added to the reaction mixture, and NBD fluorescence intensities were set to 0 at 0 s. Traces show means±s.d. of three independent experiments. (**c**) A schematic diagram of the liposome flotation assay (see details in Methods). (**d**) The Ups1–Mdm35 complex was incubated with liposomes containing PC alone (PC only) or PC/PA=80/20 (PC+PA) at pH 5.5 or pH 6.5 and binding was analysed by liposome flotation as in **c**. T, top; B, bottom. The amounts of Ups1 are shown below the gel (total Ups1 amounts were set to 100). (**e**) WT and the indicated mutant Ups1 proteins in a complex with Mdm35 were incubated with cardiolipin-containing liposomes (PC/CL=80/20) and binding was analysed as in **c**. (**f**) Mitochondria isolated from WT or *crd1*Δ cells were sonicated to form OM and IM vesicles. The vesicles were subjected to sucrose-gradient centrifugation, fractionated (left, top; right, bottom) and analysed by SDS–PAGE and immunoblotting using the indicated antibodies. (**g**) A schematic diagram of the assay for PA-extraction activities of the Ups1–Mdm35 complex from liposomes (see details in Methods). (**h**) The protein supernatant separated from the donor liposomes in **g** was subjected to gel-filtration using a Superdex 200 10/300 GL column. Absorbance at 280 and 460 nm indicating the Ups1–Mdm35 complex (blue) and NBD-PA (red), respectively, were monitored. Vo, void volume. Arrowheads indicate non-specific peaks.

On the basis of the structure of the Ups1–Mdm35 complex, we made several Ups1 mutants for *in vitro* PA transfer assays as well as PA extraction and liposome binding assays. We used the full-length Ups1–Mdm35 complex for the following functional assays. We replaced the positively charged residues Arg25 at the bottom of the pocket and Lys61 and Lys155 near the entrance of the pocket with a negatively charged Glu (R25E and K61E/K155E). We also made a Ups1 mutant lacking the Ω1 loop (lacking residues 62–71; ΔLid). In the fluorescent-based PA transfer assay, donor liposomes-containing NBD-labelled PA (NBD–PA) and rhodamine-labelled PE (Rhod-PE), which quenches NBD fluorescence, are incubated with the purified Ups1–Mdm35 mutants and acceptor liposomes without fluorescent lipids, and transfer of NBD–PA to the acceptor liposomes will lead to dequenching that is, increase in the NBD fluorescence (Fig. 5a). Wild-type (WT) Ups1–Mdm35 efficiently increased NBD fluorescence in a time-dependent manner as compared with the negative control without added proteins or acceptor liposomes (Fig. 5b, Supplementary Fig. 4a). We first confirmed that the C-terminal deletion of the Ups1–Mdm35 does not affect the PA transfer activity since Ups1ΔC5–Mdm35ΔC5 used for crystallization showed the PA transfer activity as efficient as full-length Ups1–Mdm35 (Supplementary Fig. 4b). We also confirmed that Ups1–Mdm35 specifically transfers PA but not PS (Supplementary Fig. 5), which is consistent with recent studies^20^,^21^. Both R25E and K61E/K155E mutations nearly abolished the PA transfer activities of Ups1–Mdm35, while R25K mutations without a charge alteration or reverse charge mutations of Lys6 and Lys128 in the opposite face of the Ups1 molecule (K6E/K128E) did not markedly impair the NBD fluorescence increase (Fig. 5b). Deletion of the Ω1 loop (ΔLid) also completely impaired the PA transfer activity of Ups1–Mdm35. These results indicate that conserved basic residues at the bottom and near the entrance of the pocket and the Ω1 lid play essential roles in the PA transfer activity of Ups1–Mdm35.

We next examined in which step of the PA transfer process the Arg25, Lys61, Lys155 and the Ω1 lid are important. We thus first tested binding of Ups1–Mdm35 mutants to lipid membranes by liposome floatation assays (Fig. 5c). Ups1 was previously shown to bind to liposomes containing acidic phospholipids at low pH condition (for example, pH 5.5) on dissociation from Mdm35 (ref. 20). We confirmed that, at pH 5.5, Ups1 binds to PA containing liposomes, but not to PC-only liposomes (Fig. 5d, fraction T). However, at pH 6.5, which is close to the physiological pH (6.88) of IMS of human mitochondria^35^, Ups1 did not bind to even PA-containing liposomes (Fig. 5d), but instead bind to cardiolipin-containing liposomes (Fig. 5e, WT). This suggests that the presence of cardiolipin rather than PA in the membrane is more important for stable binding of Ups1 to lipid membrane under physiological conditions. This was also confirmed *in vivo*. We isolated mitochondria from WT and *crd1*Δ cells lacking the cardiolipin synthase and prepared OM and IM vesicles by sonication. Then OM and IM vesicles were separated by sucrose density-gradient centrifugation and distribution of Ups1 was assessed by immunoblotting. Ups1 was recovered exclusively in the IM vesicles in WT mitochondria, while it was evenly distributed to the OM and IM vesicles in *crd1*Δ mitochondria lacking cardiolipin (Fig. 5f). These results again point to the important role of cardiolipin in the exclusive IM-localization of Ups1 in mitochondria.

Then we asked if the Ups1–Mdm35 mutants could bind to cardiolipin-containing liposomes at pH 6.5. Interestingly, while ΔLid, K6E/K128E, R25E and R25K mutants were recovered with cardiolipin-containing liposomes, K61E/K155E mutant could hardly bind to cardiolipin-containing liposomes (Fig. 5e). Therefore, basic residues near the entrance of the pocket of Ups1 are important for binding to lipid membranes, which may be at least partly the reason for the impaired PA transfer activity of the K61E/K155E mutant. On the other hand, the defects in the PA transfer activity observed for ΔLid and R25E Ups1 mutants should be attributed to the step(s) other than lipid-membrane binding.

We next examined whether the Ups1–Mdm35 mutants are capable of extracting PA from lipid membranes. In the PA extraction assay, NBD-PA containing liposomes loaded with 12.5% sucrose are incubated with Ups1–Mdm35, and then the protein is separated from heavier liposomes by centrifugation to monitor the shift of NBD–PA from liposomes to the protein (Fig. 5g). NBD–PA was detected by absorbance at 460 nm in the peak of absorbance at 280 nm in the gel-filtration elution profiles (Supplementary Fig. 6). The Ups1–Mdm35 complex with NBD–PA was eluted at the same elution volume as that without NBD–PA (Supplementary Fig. 6). These results suggest that, after extracting PA from liposomes on dissociation from Mdm35, Ups1 forms a ternary complex with Mdm35 and PA. When PA extraction abilities were compared among various Ups1 mutants, PA was efficiently extracted by Ups1–Mdm35 with WT, R25K and K6E/K128E mutant Ups1 (Fig. 5h). On the other hand, Ups1–Mdm35 with ΔLid, R25E and K61E/K155E mutant Ups1 failed to extract NBD–PA from liposomes (Fig. 5h). Although the K61E/K155E Ups1 mutant cannot extract NBD-PA from liposomes probably due to its reduced ability to bind to liposomes (Fig. 5e), the ΔLid and R25E Ups1 mutants, which are normal in binding to lipid membranes, lack the ability to stably accommodate NBD–PA in the pocket.

### Mdm35 dissociation aids liposome binding and PA transfer

**Figure 6:**
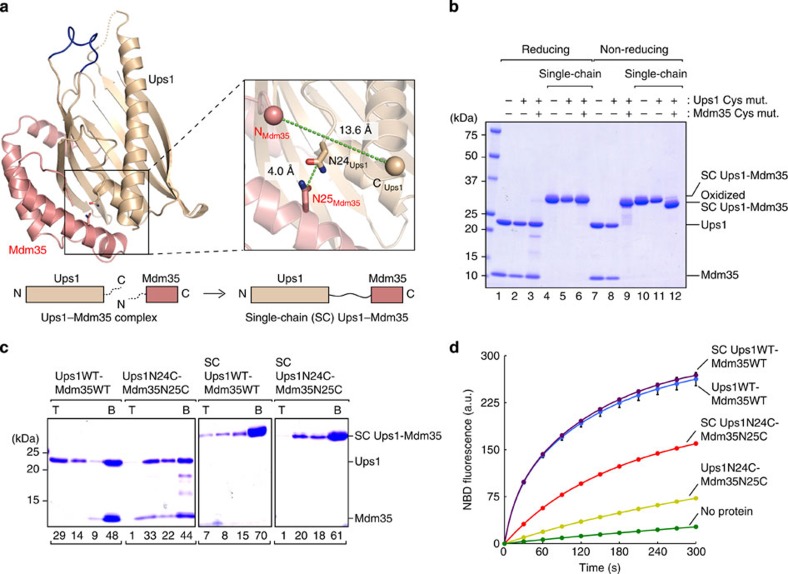
Lipid binding and PA transfer activities of single-chain Ups1–Mdm35 derivatives. (**a**) Magnified view around Asn24 and the C-terminal Glu169 of Ups1 (the C-terminal one residue is disordered in Ups1ΔC5) and Asn25 and the N-terminal Asn3 of Mdm35 (the N-terminal two residues are disordered in Mdm35ΔC5). Cα atoms of Glu169 of Ups1 and Asn3 of Mdm35 are shown as sphere. Distances between the C terminus of Ups1 and the N terminus of Mdm35 and between C_γ_ atoms of Asn24 in Ups1 and Asn25 in Mdm35 are indicated. Schematic diagrams of the Ups1ΔC5– Mdm35ΔC5 hetero-dimeric complex and single-chain Ups1–Mdm35 consisting of full-length Ups1 (1–175) fused to full-length Mdm35 (1–86) are shown at the bottom. (**b**) Ups1 with and without N24C mutation and Mdm35 with and without N25C mutation in separate chains or a single-chain polypeptide were analysed by SDS–PAGE with (Reducing) or without (Non-reducing) β-mercaptoethanol and CBB staining. (**c**) Ups1WT–Mdm35WT, Ups1N24C–Mdm35N25C, single-chain Ups1WT–Mdm35WT and single-chain Ups1N24C–Mdm35N25C were incubated with cardiolipin-containing liposomes (PC/CL=80/20) and binding was analysed at 25 °C and pH 6.5 by flotation assay as in Fig. 5d. T, top; B, bottom. The amounts of Ups1 are shown below the gel (total Ups1 amounts were set to 100). (**d**) PA transfer activities of Ups1WT–Mdm35WT, Ups1N24C–Mdm35N25C, single-chain Ups1WT–Mdm35WT and single-chain Ups1N24C–Mdm35N25C were analysed as in Fig. 5b. Traces show means±s.d. of three independent experiments.

Mdm35 was previously shown to dissociate from Ups1 on binding to lipid membranes^20^, however, it is not clear if this Mdm35 dissociation is prerequisite for lipid-membrane binding of Ups1. To address this question, we attempted to make a covalently tethered Ups1–Mdm35 complex. Since the crystal structure of the Ups1–Mdm35 complex indicates that the C terminus of Ups1 is close to the N terminus of Mdm35 (13.6 Å, Fig. 6a), we tandemly fused Ups1 to Mdm35 to make a single-chain mutant so that Ups1 and Mdm35 are no longer able to dissociate. We also replaced Asn24 of Ups1 and Asn25 of Mdm35, which are closely positioned in the Ups1–Mdm35 binding interface, with Cys to stabilize the Ups1–Mdm35 contact by intermolecular disulfide bridge formation (Fig. 6a). As expected, separately expressed Ups1N24C and Mdm35N25C were detected as a single band whose size on a SDS–polyacrylamide gel electrophoresis (SDS–PAGE) gel (non-reducing condition) is the same as the single-chain variant of Ups1–Mdm35, indicating Cys residues at positions 24 of Ups1 and 25 of Mdm35 are close to each other to form a disulfide bond (Fig. 6b, lanes 3 and 9). A disulfide bond was also formed when the Ups1N24C and Mdm35N25C domains are synthesized as a single polypeptide because the band of the single-chain protein on a SDS–PAGE gel shifted upward on reducing treatment (Fig. 6b, lanes 6 and 12). CD spectra showed that the single-chain fusion and Cys replacement do not affect overall folding of the Ups1–Mdm35 complex (Supplementary Fig. 7a). We also examined the effects of the Cys replacement on the stability of the Ups1N24C–Mdm35N25C complex by monitoring the exchange of prebound Mdm35N25C with free GST–Mdm35 as in Fig. 2d, with and without intermolecular disulfide bridge formation (that is, pretreated with DTT) (Supplementary Fig. 7b). Ups1N24C–Mdm35N25C with the intermolecular disulfide bond prevents dissociation of Mdm35N25C from Ups1N24C, thereby inhibiting exchange of Mdm35N25C with GST–Mdm35, that is, copurificaiton of Ups1 with GST–Mdm35, as expected. As a control, DTT pretreated Ups1N24C–Mdm35N25C lacking the intermolecular disulfide bond was efficiently copurified with GST–Mdm35. Interestingly, single-chain Ups1–Mdm35 and DTT pretreated single-chain Ups1N24C–Mdm35N25C lacking the disulfide bond between Ups1 and Mdm35 were not efficiently copurified with GST–Mdm35 as compared with WT Ups1–Mdm35, suggesting that the single-chain fusion itself stabilizes the association of Ups1 with Mdm35.

We then tested the ability of the disulfide linked and/or single-chain variants of Ups1–Mdm35 to bind to cardiolipin-containing liposomes and to transfer PA between liposomes. The disulfide linked and/or single-chain variants of Ups1 did not bind to cardiolipin-containing liposomes efficiently (Fig. 6c), but pretreatment of Ups1N24C–Mdm35N25C with 5 mM DTT partly recovered Ups1N24C binding to cardiolipin-containing liposomes (Supplementary Fig. 7c). These results suggest that the dissociation of the Ups1–Mdm35 complex is important for stable binding of Ups1 to the lipid membrane. Supporting this notion, interactions between the Ups1–Mdm35 complex and cardiolipin-containing liposomes were diminished in the presence of excess amounts of free Mdm35, which is expected to shift the equilibrium of Ups1 from the free form to the Mdm35-bound form (Supplementary Fig. 8a).

Then, we performed PA transfer assays and found that disulfide-bond fixation of the Ups1–Mdm35 contact impairs the PA transfer activity as compared with WT Ups1–Mdm35 (Fig. 6d). The single-chain fusion, which stabilizes the Ups1–Mdm35 association (Fig. 6d) or the presence of excess amounts of free Mdm35 (Supplementary Fig. 8b–d) did not affect the PA transfer or PA extraction activities, suggesting that dynamic transient dissociation of Ups1–Mdm35 is sufficient for PA transfer and extraction. Indeed, PA transfer (Supplementary Fig. 9a) and PA extraction (Supplementary Fig. 9b) activities of the disulfide linked Ups1–Mdm35 were restored after treatment with DTT, although Cys replacement itself moderately decreased PA extraction activities (Supplementary Fig. 9b). We also monitored the step of PA release from the Ups1–Mdm35 variants on addition of acceptor liposomes, by using the Ups1–Mdm35 variants loaded with PA from donor liposomes (Supplementary Fig. 10a). PA release became slow when Ups1 and Mdm35 variants were covalently linked by the disulfide bond (Supplementary Fig. 10b,c), and these PA release activities were restored after treatment with DTT (Supplementary Fig. 10d). Taken together, we conclude that dynamic dissociation and association of Ups1 and Mdm35 contribute to efficient lipid membrane binding, PA extraction, PA release and thereby PA transfer, although stable lipid-membrane binding is more sensitive to the Ups1–Mdm35 complex stabilization than PA transfer activities.

## Discussion

The fundamental question concerning lipid trafficking is how hydrophobic lipid molecules, which normally reside in membranes, are transported between the membranes by crossing the aqueous divide. Here we focused on the Ups1–Mdm35 system, which mediates PA transfer from the OM to IM within mitochondria, and determined its X-ray crystal structures with and without a substrate DLPA. On the basis of the determined structures of Ups1–Mdm35, we introduced several mutations in Ups1 and analysed their functional consequences mainly by using *in vitro* assays, as yeast cells can often tolerate defects in lipid transport/synthetic pathways by activating other pathways^4^,^8^,^20^.

Ups1 in the complex with Mdm35 was shown to be structurally similar to START domains found in several lipid-transfer proteins^28^,^29^,^30^,^31^,^32^. Lipid recognition mechanisms are partly shared by Ups1 and other START domains with known structures. The head group of lipid is recognized by hydrophilic residues at the bottom of the pocket, whereas the tail group of lipid is recognized by hydrophobic residues in the inner wall of the pocket (Supplementary Fig. 11). Ups1 also has a deep pocket that can accommodate its ligand PA molecule and the extended Ω1 loop (Figs 3a and 4c), and the inner wall of the pocket is amphiphilic in nature and rich in positively charged residues. In particular, Ups1 has Arg25 at the bottom and Lys61 and Lys155 near the rim of the pocket entrance, all of which were shown to be important for PA transfer between liposomes *in vitro* (Fig. 5b). Arg25 may be involved in recognition of the phosphate group of PA for substrate specificity, and Lys61 and Lys155 may mediate efficient binding of Ups1 to the acidic phospholipid including cardiolipin in the membrane (Figs 4c and 5e). In the structure of Ups1, the Ω1 lid appears to prevent PA from entering or leaving the pocket (Fig. 3a). Although the Ω1 lid is not involved in lipid-membrane binding (Fig. 5e), the Ω1 loop exhibits high B-factors (Fig. 3d), meaning that the Ω1 lid is rather flexible as compared with the rest of the Ups1 molecule. Besides, we found that the Ω1 lid could take an alternative open conformation that provides a wide space rendering the pocket entrance accessible to PA (Supplementary Fig. 3). Therefore, the lid may function as the gate for PA entry into and PA exit from the pocket. Indeed, ΔLid and R25E mutants of Ups1 are defective in PA extraction from liposomes (Fig. 5h). Since the size of the entrance of the pocket is about 9 Å in diameter, which is too narrow to allow two acyl tails of PA to pass through freely, PA binding to the pocket may require opening of the entrance as well as a swing of the Ω1 lid away from the pocket entrance (as observed in Supplementary Fig. 3). The closed Ω1 lid may be important for preventing the bound PA molecules from slipping off the pocket, as well.

The structure of the Ups1–Mdm35 complex revealed that Mdm35 embraces Ups1 through multiple conserved hydrophobic interactions involving three α-helices of Mdm35 (Figs 1c and 2a), which can explain the previous observation that Mdm35 facilitates import of Ups proteins as a possible trap in the IMS^26^,^27^. Mdm35 and cardiolipin are also important for regulating stable Ups1 binding to membranes (the IM *in vivo*) at physiological pH (Fig. 5d,e). Prevention of dissociation of Mdm35 from Ups1 by disulfide bond tethering reduced the abilities of Ups1 to bind to cardiolipin-containing liposomes and to transfer PA between liposomes, although these abilities were not lost completely (Fig. 6c,d). Dissociation of Mdm35 from Ups1 would lead to exposure of the hydrophobic Mdm35-binding region on Ups1, which may facilitate interactions with hydrophobic membranes. Therefore, dissociation of Mdm35 from Ups1 could achieve efficient PA transfer through efficient membrane binding.

In conclusion, the determined structures of the Ups1–Mdm35 complex with and without PA offer a structural basis for further understanding of the molecular mechanism of phospholipid transfer between the mitochondrial OM and IM.

## Methods

### Plasmids

To construct expression plasmids for GST fused to the N terminus of full-length Mdm35 or Mdm35ΔC5, the corresponding genes were amplified by PCR and cloned into pGEX-6p-1 (GE Healthcare). To construct coexpression plasmids for N terminally hexahistidine (His_6_) tagged full-length Ups1 and GST-Mdm35, or His_6_-Ups1ΔC5 and GST-Mdm35ΔC5, the corresponding genes were amplified by PCR and cloned into pETDuet-1 (Novagen). Mutations for the described amino-acid substitutions were introduced by PCR-based site-directed mutagenesis. The expression plasmids for the N-terminally His_6_ tagged single-chain Ups1–Mdm35 and deletion mutants of Ups1 or Mdm35 were constructed by inverse PCR. To generate the pRS314/*GAL1*-Mdm35 plasmid, the Mdm35 ORF was amplified by PCR, digested with BamHI and XhoI, and inserted into the BamHI/XhoI site between the *GAL1* promoter and *CYC1* terminator in the *CEN*–*TRP1* plasmid, pRS314. Mutations for the described amino-acid substitutions were introduced by PCR-based site-directed mutagenesis. The resulting constructs were sequenced to confirm their identities. We refer to Ups1ΔC5 and Mdm35ΔC5 as Ups1 and Mdm35 below.

### Protein expression and purification

The target proteins were expressed in *E. coli* SHuffle T7 cells (NEB) cultured in LB medium. After addition of 0.1 mM isopropyl-D-thiogalactoside, the cells were cultured at 20 °C for 20 h, and were disrupted by sonication. GST–Mdm35 derivatives were affinity purified by a glutathione-Sepharose 4B (GS4B) column (GE Healthcare). For crystallization of Mdm35, the GST tag was cleaved off with PreScission protease (GE Healthcare) and removed by a GS4B column. The obtained proteins were further purified by a HiLoad 26/60 Superdex 200 PG column (GE Healthcare) with elution buffer of 20 mM Tris-HCl pH 7.5 and 150 mM NaCl. The His_6_-tagged Ups1–Mdm35 complexes were affinity purified by a Ni–NTA agarose column (QIAGEN). The obtained proteins were purified by a HiLoad 26/60 Superdex 200 PG column (GE Healthcare), and when necessary, further purified by a SP sepharose column. The SeMet-labelled proteins were prepared from *E coli* SHuffle T7 (NEB) cells cultured in minimal media containing 25 mg l^−1^ SeMet. Several PA species with different acyl chains; POPA (840857C), DPPA (830855X), DMPA (830845X), DLPA (840635P), DDPA (830843C) and DHPA (830841C) were obtained from Avanti Polar Lipids and used for preparation of the Ups1–Mdm35–PA complex. 0.2 mM Ups1–Mdm35 complex was incubated with 1.0 mM DLPA (12:0-12:0; Avanti Polar Lipids) in 20 mM MES–NaOH pH 5.5, 150 mM NaCl and 2.0 mM dodecylmaltoside (DOJINDO) micelles at room temperature for 1 h. After lowering the dodecylmaltoside concentration below the critical micelle concentration, the Ups1–Mdm35–PA complex was then affinity purified by a Ni–NTA agarose column, and further purified by a HiLoad 26/60 Superdex 200 PG column. Among several PA species, the Ups1–Mdm35 complex with DLPA grew crystals suitable for structural analyses. We refer to the Ups1–Mdm35-DLPA complex as the Ups1–Mdm35–PA complex below.

### Crystallization

All crystallization trials were performed using the sitting drop vapour diffusion method at 20 °C. For crystallization of Mdm35 or SeMet-labelled Mdm35, drops (0.5 μl) of ∼65 mg ml^−1^ protein in 20 mM Tris-HCl pH 7.5, and 150 mM NaCl were mixed with reservoir solution consisting of 1.8 M ammonium sulfate, 0.1 M MES–NaOH pH 6.5 and 10 mM CoCl_2_, and equilibrated against 70 μl of the same reservoir solution by vapour diffusion. For crystallization of the Ups1–Mdm35 complex, drops (0.5 μl) of 32 mg ml^−1^ protein in 20 mM Tris-HCl pH 7.5, and 150 mM NaCl were mixed with the reservoir solution consisting of 0.2 M ammonium citrate tribasic, pH 7.0 and 20% polyethylene glycol 3,350, and equilibrated against 70 μl of the same reservoir solution by vapour diffusion. For crystallization of the SeMet-labelled Ups1–Mdm35 complex, drops (0.5 μl) of 20 mg ml^−1^ protein in 20 mM Tris-HCl pH 7.5 and 150 mM NaCl were mixed with the reservoir solution consisting of 0.1 M Tris-HCl pH 8.0, and 31% polyethylene glycol monomethyl ether 2,000, and equilibrated against 70 μl of the same reservoir solution by vapour diffusion. For crystallization of the Ups1–Mdm35–PA complex, drops (0.5 μl) of 32 mg ml^−1^ protein in 20 mM Tris-HCl pH 7.5 and 150 mM NaCl were mixed with reservoir solution consisting of 0.1 M HEPES–NaOH pH 7.5, 10% polyethylene glycol 6,000 and 5% (+/−)-2-methyl-2,4-pentanediol and equilibrated against 70 μl of the same reservoir solution by vapour diffusion.

### X-ray crystallography

Diffraction data were collected with an ADSC Quantum 315 CCD detector at a SPring-8 beam line BL38B1 for Mdm35, SeMet-labelled Mdm35, and the SeMet-labelled Ups1–Mdm35 complex, with an ADSC Quantum 270 CCD detector at a Photon Factory beam line NE3A for the Ups1–Mdm35 complex, and with an ADSC Quantum 210r CCD detector at a Photon Factory beam line NW12A for the Ups1–Mdm35–PA complex. All diffraction data were processed with HKL2000 (ref. 36). The initial phasing of Mdm35 was performed by the multiwavelength anomalous dispersion method with the peak, inflection and high-remote data of the SeMet-labelled crystal. After two selenium sites were identified and the initial phases calculated, density modification and automated model building were performed with Phenix^37^. The obtained model was transferred to the native crystal data of Mdm35. The initial phasing of the Ups1–Mdm35 complex was performed by the single-wavelength anomalous dispersion method with the peak data of the SeMet-labelled crystal. After six selenium sites were identified and the initial phases calculated, density modification and automated model building were performed with Phenix. The obtained model was transferred to the native crystal data. The structure of the Ups1–Mdm35–PA complex was determined by the molecular replacement method with MOLREP (ref. 38) in CCP4 (ref. 39), for which the Ups1–Mdm35 complex structure was used as a search model. For all the structures, further model building was performed manually with COOT (ref. 40), and crystallographic refinement with CNS (ref. 41) (Table 1, Supplementary Fig. 12). Procheck^42^ was used to assess the quality and geometry of the structural models. Ramachandran statistics: Mdm35, 91.8% most favored, 8.2% additionally allowed and no disallowed rotamers; Ups1–Mdm35, 92.2% most favored, 7.8% additionally allowed and no disallowed rotamers; Ups1–Mdm35–PA, 80.7% most favoured, 17.5% additionally allowed, 1.9% generously allowed and no disallowed rotamers.

### Affinity copurification assay

GST-Mdm35 mutants (10 μM) in 20 mM Tris-HCl pH 7.5 and 150 mM NaCl and 10 μM of His_6_–Ups1–Mdm35 in 20 mM the same buffer were mixed and incubated with 50 μl of GS4B resin at 37 °C for 15 min. The resin was washed three times with 500 μl of 20 mM Tris-HCl pH 7.5, 150 mM NaCl and proteins were eluted with 50 μl 10 mM glutathione in 50 mM Tris-HCl pH 8.0. The eluted proteins were subjected to SDS–PAGE and immunoblotting with the anti-His tag antibody (GE Healthcare).

### Yeast strains and media

Yeast strains used in this study are summarized in Table 2. Standard protocols were used for yeast manipulation^43^. Cells were grown in YPD (1% yeast extract, 2% polypeptone and 2% glucose) media, SCD (0.67% yeast nitrogen base without amino acids, 0.5% casamino acid and 2% glucose) and SCGal+succrose (0.67% yeast nitrogen base without amino acids, 0.5% casamino acid, 2% galactose and 2% sucrose) media with appropriate supplements.

### Liposomes

Phospholipids: POPC (1-palmitoyl-2-oleoyl-*sn*-glycero-3- phosphocholine, 850457C), POPE (1-palmitoyl-2-oleoyl-*sn*-glycero-3- phosphoethanolamine, 850757C), POPA, *E*. *coli* cardiolipin (841199), 18:1-12:0 NBD-PA (1-oleoyl-2-(12-[(7-nitro-2-1,3- benzoxadiazol-4-yl)amino]dodecanoyl)-*sn*-glycero-3-phosphate, 810176C), 18:1-12:0 NBD-PS (1-oleoyl-2-(12-[(7-nitro-2-1,3-benzoxadiazol-4-yl)amino]dodecanoyl) -*sn*-glycero-3-phosphoserine, 810195C), and Egg Liss-Rhod-PE (L-α- phosphatidylethanolamine-*N*-(lissamine rhodamine B sulfonyl), 810146C) were obtained from Avanti Polar Lipids. Lipids in stock solutions in chloroform were mixed at the desired molar ratio, and the solvent was evaporated. The lipid film was hydrated in appropriate buffer. The lipid suspension was incubated at room temperature for 30 min and extruded 20 times through polycarbonate 0.1-μm filter using a mini-extruder (Avanti Polar Lipids).

### Liposome flotation assay

Liposome flotation assay was performed as described previously^20^ with several modifications. Two millimolar liposomes (POPC/*E. coli* cardiolipin=80/20) were incubated with 5 μM Ups1–Mdm35 or its mutant in 100 μl of flotation buffer (20 mM PIPES-NaOH pH 6.5, 100 mM NaCl and 1 mM EDTA) at 25 °C for 10 min. 200 μl of ice-cold flotation buffer with 60% sucrose was added to the sample after the incubation. The resulting liposome/protein mixture was put into a 5 ml of an ultracentrifuge tube and overlaid with 2 ml of flotation buffer with 30% sucrose, 2 ml of 10% sucrose and then 1 ml of flotation buffer without sucrose. After centrifugation at 200,000 *g* for 1.5 h, 1,400 μl of fractions were collected from the top and the proteins were concentrated by TCA precipitation. The proteins were analysed by SDS–PAGE and CBB staining.

### PA transfer assay

PA-transfer activities of the Ups1–Mdm35 complex for Ups1 mutants were measured by the fluorescent dequenching assay as described previously^21^ with several modifications. Donor liposomes (6.25 μM; POPC/POPE/Egg Liss-Rhod-PE /18:1-12:0 NBD-PA=50/40/2/8) were incubated with acceptor liposomes (25 μM; POPC/POPE/POPA=50/40/10) in the presence or absence of 20 nM purified Ups1–Mdm35 complex in 2 ml of assay buffer (20 mM Tris-HCl pH 7.5, 150 mM NaCl and 2 mM EDTA) at 25 °C. The NBD fluorescence was monitored by a FP-6500 spectrofluorometer (Jasco).

### PA extraction assay using gel-filtration chromatography

Ups1–Mdm35 (10 μM) complex for Ups1 mutants in 20 mM Tris-HCl, pH 7.5 and 150 mM NaCl were incubated with 200 μM donor liposomes (POPC/POPE/18:1–12:0 NBD-PA=50/40/10) containing 12.5% sucrose in 20 mM Tris-HCl pH 7.5 and 150 mM NaCl. Liposomes were collected by centrifugation at 150,000 *g* for 1 h, and the supernatant was subjected to gelfiltration using a Superdex 200 10/300 GL (GE healthcare) column.

### Fluorescent-based PA extraction assay

Donor liposomes (6.25 μM; POPC/POPE/Egg Liss-Rhod-PE /18:1–12:0 NBD-PA=50/40/2/8) were incubated with or without of 200 nM purified Ups1–Mdm35 complex in 2 ml of assay buffer (20 mM Tris-HCl pH 7.5, 150 mM NaCl and 2 mM EDTA) at 25 °C. The NBD fluorescence was monitored by a FP-6500 spectrofluorometer.

### Fluorescent-based PA release assay

Donor liposomes (6.25 μM; POPC/POPE/Egg Liss-Rhod-PE /18:1–12:0 NBD-PA=50/40/2/8) were incubated with or without of 200 nM purified Ups1–Mdm35 complex in 2 ml of assay buffer (20 mM Tris-HCl pH 7.5, 150 mM NaCl and 2 mM EDTA) at 25 °C for 300 s., then acceptor liposomes (25 μM; POPC/POPE/Egg Liss-Rhod-PE/POPA=50/40/2/8) were added. We adjusted the protein concentrations between 200 and 1,100 nM to achieve similar NBD-PA loading to the proteins (as monitored by NBD fluorescence after 300 s incubation) to directly compare the subsequent PA release from the proteins on addition of the acceptor liposomes. The NBD fluorescence was monitored by a FP-6500 spectrofluorometer.

### Seperation of the outer and inner membrane vesicles

Mitochondrial outer and inner membrane vesicles were separated essentially as described previously^44^. Briefly, 10 mg of WT or *crd1Δ* mitochondria expressing Ups1-FLAG were suspended in 3.7 ml of EM buffer (1 mM EDTA, 20 mM MOPS-KOH, pH 7.2) and kept on ice for 30 min to open the OM by osmotic swelling. The resulting ‘mitoplasts' were further incubated for 10 min on ice after addition of 1.28 ml of 60% sucrose in EM buffer. The sample was then sonicated thoroughly until the mitoplast suspension became clear. After removal of residual intact mitochondria by centrifugation at 18,800 r.p.m. in the SW55Ti rotor (Beckman) for 20 min at 4 °C, OM and IM vesicles in the supernatant were precipitated by centrifugation at 47,000 r.p.m. in the SW55Ti rotor for 40 min at 4 °C. The resulting pellet was re-suspended in 200 μl of EM buffer with 10 mM NaCl, placed onto 5 ml of the sucrose step gradient consisting of 1.25 ml of 26, 31, 35 and 40% sucrose in EM buffer, and then centrifuged at 39,000 r.p.m. for 16 h in the SW55Ti rotor. Three-hundred and twenty microlitres each of fractions was collected from the top, and 20 μl of each fraction was used for immunoblotting.

### Immunoblotting

Primary antibodies against Tom70, Tom22, Tim23, Tom40, Psd1 and Mdm35 were raised in rabbits in our laboratory. The antisera were used in 1:2,000 dilutions for immunoblotting. Anti-FLAG and anti-Histidine tag antibodies purchased from Sigma and GE Healthcare, respectively, were used in 1:2,000 dilutions. Proteins were visualized with fluorophore-conjugated secondary antibodies, Goat anti-Rabbit or Mouse IgG (H+L) Secondary Antibody, Cy 5 or Alexa Fluor 488 conjugate (Life Technologies) and the signals were analysed with a Storm 860, Typhoon 9200 (GE Healthcare) or PharosFX Plus Molecular Imager (Bio-Rad).

### CD measurements

Circular dichroism (CD) spectra of proteins (5 μM) in CD buffer (20 mM Tris-HCl pH 7.5 and 150 mM NaCl) were recorded at 25 °C on a J-720 spectropolarimeter (Jasco), using a cell with a path length of 0.2 cm.

### Miscellaneous

Original images of gels and blots used in this study can be found in Supplementary Fig. 13.

## Additional information

**Accession codes:** Atomic coordinates and structure factors files have been deposited in the Protein Data Bank under accession codes 4YTV (Mdm35), 4YTW (the Ups1–Mdm35 complex) and 4YTX (the Ups1–Mdm35 complex with PA).

**How to cite this article:** Watanabe, Y. *et al*. Structural and mechanistic insights into phospholipid transfer by Ups1–Mdm35 in mitochondria. *Nat. Commun.* 6:7922 doi: 10.1038/ncomms8922 (2015).

## Supplementary Material

Supplementary Information Supplementary Figures 1-13

## Figures and Tables

**Table 1:** Data collection, phasing and refinement statistics.

	**NativeMdm35**	**SeMet labelledMdm35**			**NativeUps1–Mdm35**	**SeMet labelledUps1-Mdm35**	**NativeUps1–Mdm35-PA**
Data collection							
Space group	*R*32	*R*32			*P*2_1_	*P*2_1_	*C*2
Cell dimensions							
*a*, *b*, *c* (Å)	59.14, 59.14, 123.28	59.09, 59,09, 123.13			42.75, 71.75, 87.61	42.75, 38.16, 70.22	208.64, 154.67, 99.01
*α*, *β*, *γ* (°)	90.0, 90.0, 120.0	90.0, 90.0, 120.0			90.0, 95.02, 90.0	90.0, 97.86, 90.0	90.0, 104.42, 90.0
Data set		*Peak*	*Inflection*	*Remote*			
Wavelength (Å)	1.00000	0.97919	0.97932	0.96426	1.00000	0.97912	1.00000
Resolution range (Å)	50.0–1.45	50.0–1.50	50.0–1.50	50.0–1.50	50.0–1.40	50.0–2.80	50.0–3.20
Outer shell (Å)	1.48–1.45	1.55–1.50	1.55–1.50	1.55–1.50	1.42–1.40	2.90–2.80	3.26–3.20
*R*_merge_	0.075 (0.624)	0.057 (0.480)	0.055 (0.526)	0.056 (0.534)	0.077 (0.824)	0.145 (0.391)	0.123 (>1)
*R*_pim_	0.017 (0.150)	0.012 (0.104)	0.017 (0.167)	0.017 (0.169)	0.031 (0.355)	0.033 (0.090)	0.050 (0.387)
*I/σI*	68.5 (4.6)	89.7 (6.9)	63.3 (4.0)	68.0 (7.4)	41.6 (2.2)	26.3 (7.8)	19.4 (2.5)
Completeness (%)	100.0 (100.0)	98.6 (97.4)	98.6 (97.5)	98.8 (98.1)	99.8 (99.5)	99.1 (98.7)	100.0 (100.0)
Redundancy	20.9 (18.0)	22.9 (22.1)	11.4 (10.8)	11.5 (11.5)	7.3 (6.1)	21.7 (19.1)	7.7 (7.7)
CC_1/2_ high resolution shell	0.955	0.977	0.944	0.939	0.799	0.977	0.762
							
Refinement							
Resolution (Å)	50.0–1.45				50.0–1.40		50.0–3.20
No. reflections	14,681				96,066		47,542
*R*/*R*_free_	0.196/0.211				0.220/0.236		0.251/0.300
No. atoms							
Protein	542				3,869		15,012
Ligand/ion	7				—		72
water	72				540		—
*B*-factors (Å^2^)							
Protein	21.5				22.3		94.8
Ligand/ion	41.2				—		59.8
water	31.6				30.6		—
R.m.s. deviations							
Bond lengths (Å)	0.004				0.005		0.014
Bond angles (°)	1.1				1.1		1.7

One crystal each was used for the collection of Mdm35, the Ups1–Mdm35 complex, or the Ups1-Mdm35-DLPA complex data for structure refinement. Statistic values for the highest resolution shell are shown in parentheses. Rpim is the precision-indicating merging R factor.

**Table 2:** Yeast strains used in this study.

**Strain**	**Genotype**	**Source**
YHS0089	*MATa his3 leu2 lys2 trp1 ura3 UPS1-FLAG-CgHIS3 mdm35Δ::KanMX4*	26
YHS0090	*MATa his3 leu2 lys2 trp1 ura3 UPS2-FLAG-CgHIS3 mdm35Δ::KanMX4*	26
Ups1-FLAG	*MATa his3 leu2 lys2 trp1 ura3 UPS1-FLAG-KanMX4*	This study
Ups1-FLAG/crd1Δ	*MATa his3 leu2 lys2 trp1 ura3 UPS1-FLAG-kanMX6 crd1Δ::HIS3*	This study
