# Supplementary material for: Structural and mechanistic insights into phospholipid transfer by Ups1–Mdm35 in mitochondria
Source: Nat Commun. 2015 Aug 3;6:7922. doi: 10.1038/ncomms8922 (PMC4532887; doi:10.1038/ncomms8922)
Supplement: Supplementary Information — Supplementary Figures 1-13 [file ncomms8922-s1.pdf]

Supplementary Information

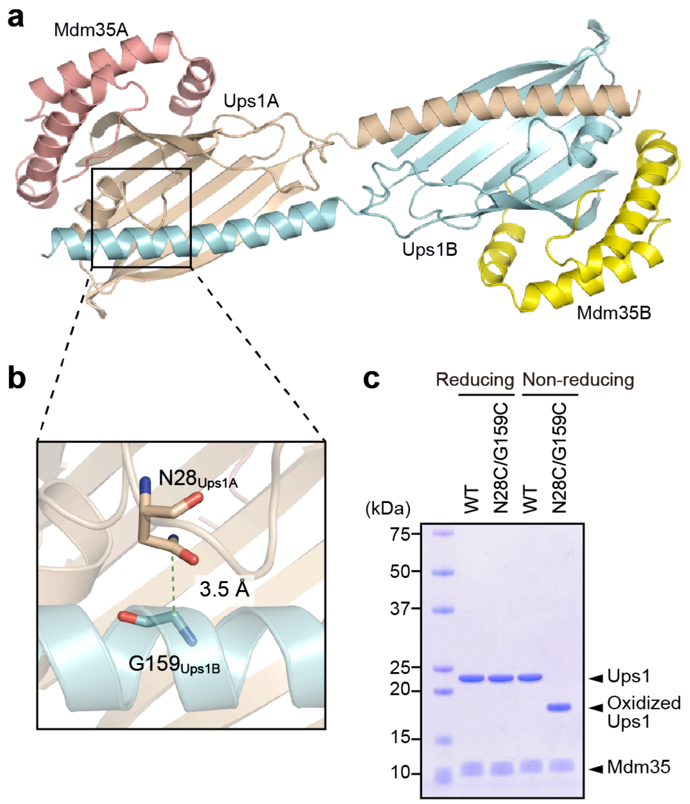

Supplementary Figure 1 | The domain-swapped dimer of Ups1 observed in crystal. (**a**) The dimeric form of the Ups1–Mdm35 complex. Ups1A, Ups1B, Mdm35A and Mdm35B in the asymmetric unit are colored in light brown, cyan, pink and yellow, respectively. (**b**) Magnified view of Asn28 in Ups1A and Gly159 in Ups1B. Distance between C_γ atom of Asn28 in Ups1A and C_α atom of Gly159 in Ups1B are indicated. (**c**) The N28C/G159C mutant was analyzed by SDS-PAGE with (Reducing) or without (Non-reducing) β-mercaptoethanol and CBB staining.

```
          1       10        20        30        40        50
                        *   *   *               *  *        *    *
Ups1      MVLLHKSTHIFPTDFASVSRAFFNRYPNPYSPHVLSIDTISRNVDQEG-NLRTTRLLKKS 59
Ups2      -MKLFQNSYDFNYPWDQVTAANWKKYPNEISTHVIAVDVLRRELKDQGKVLVTERLITVK 59

         60        70        80        90       100       110
                           * * *  * *       * * *          *
Ups1      GKLPTWVKPFLRGITETWIIEVSVVNPANSTMKTYTRNLDHTGIMKVEEYTTY---QFDS 116
Ups2      QGVPKWIMMMLGGTNMSHVREVSVVDLNKKSLTMRSCNLTMCNLLKVYETVTYSPHPDDS 119

           120       130       140       150       160       170

Ups1      ATSSTIADSRVKFSSGFNMGIKSKVEDWSRTKFDENVKKSRMGMAFVIQKLEEARNPQF- 175
Ups2      ANKTLFQQEAQITAYGSIRKLCNKMEDWSVQRFCENAKKGKMGFDAVLQVFSENWEKHVD 179


Ups1      ---------------------------------------------------
Ups2      DLSNQLVSKVNETMEDVKISAGTLLKGTERSGRTILQQNIDLFRDAYNHEN 230
```

Supplementary Figure 2 | Sequence alignment of *Saccharomyces cerevisiae* Ups1 and Ups2. Conserved residues are shaded in black, and type-conserved residues in grey. Residues marked with asterisks are in the proximity (≤ 4.5Å) of Mdm35 in the Ups1–Mdm35 structure and likely involved in the interaction with Mdm35 in Fig. 2a.

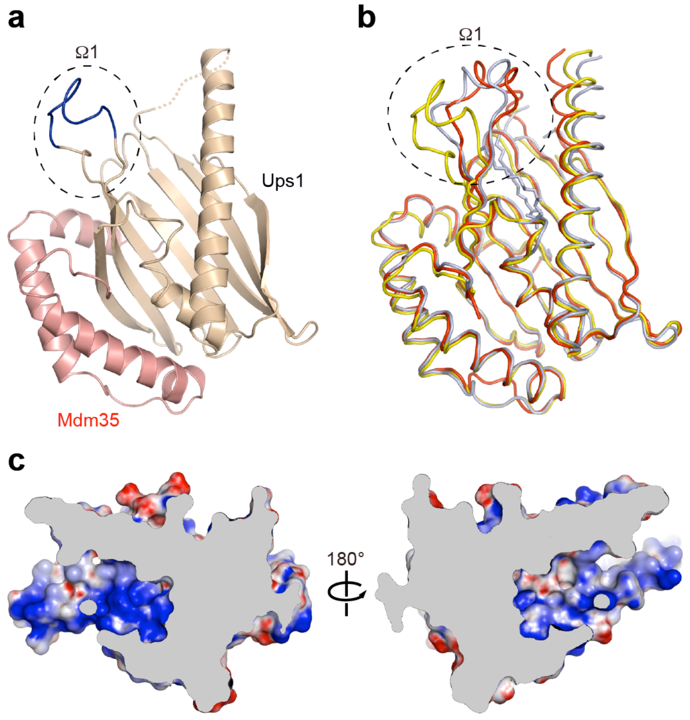

Supplementary Figure 3 | Structure of the Ups1–Mdm35 complex with the open lid. (**a**) A ribbon diagram of Ups1–Mdm35 with the open Ω1 lid. (**b**) Superposition of the apo-form (red), PA-bound form (light blue) and open-lid form (yellow) of Ups1–Mdm35. (**c**) Cutaway representation of the Ups1–Mdm35 complex with the open lid at the level of the pocket, colored according to the surface electrostatic potential.

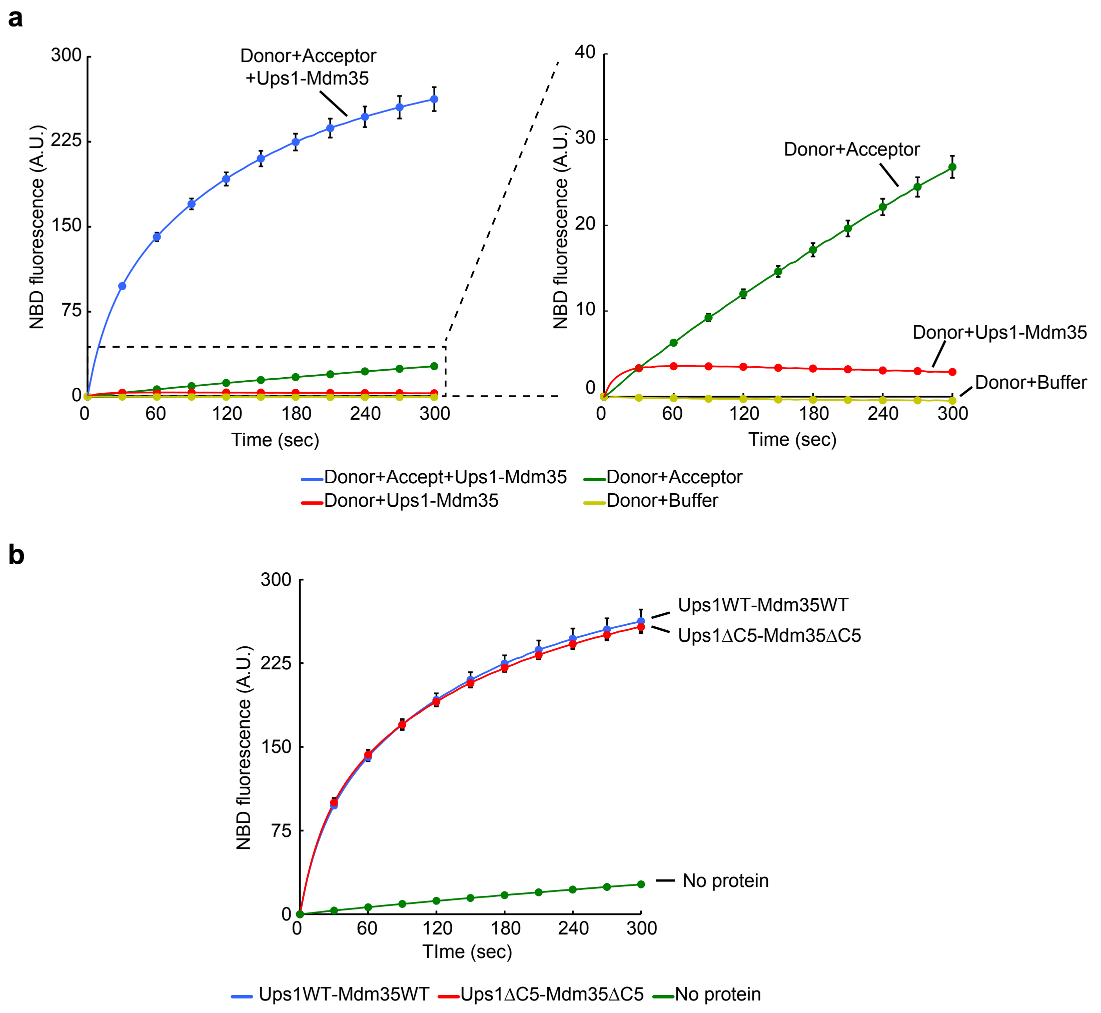

Supplementary Figure 4 | PA transfer assay. (**a**) Donor liposomes (6.25 μM; POPC/POPE/Egg Liss-Rhod-PE/18:1-12:0 NBD-PA = 50/40/2/8) were incubated with 20 nM purified Ups1–Mdm35 complex and/or acceptor liposomes (25 μM; POPC/POPE/POPA = 50/40/10) in assay buffer (20 mM Tris-HCl pH 7.5, 150 mM NaCl and 2 mM EDTA) at 25°C. NBD fluorescence was monitored as shown in Fig. 5c. At 0 sec, the protein or buffer was added to the reaction mixture, and NBD fluorescence intensities were set to 0 at 0 sec. Traces show means ± SD of three independent experiments. (**b**) PA transfer activities of the full-length Ups1–Mdm35 complex (Ups1WT–Mdm35WT) and Ups1ΔC5–Mdm35ΔC5 complex were measured at 25 °C as in **a**. Traces show means ± SD of three independent experiments.

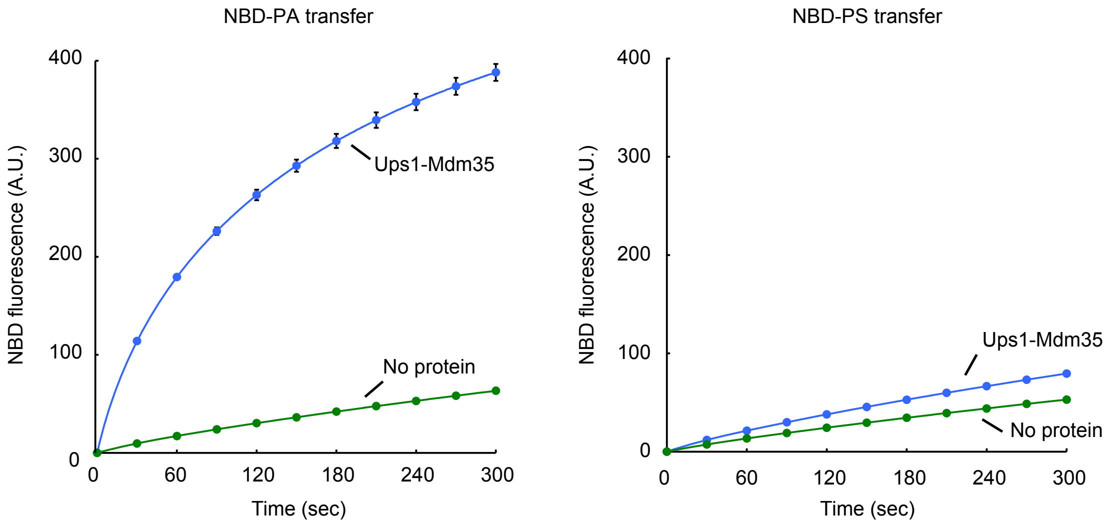

Supplementary Figure 5 | PS transfer activity of Ups1–Mdm35. Left panel: PA transfer assay. Donor liposomes (6.25 µM; POPC/POPE/Egg Liss-Rhod-PE/18:1-12:0 NBD-PA = 50/40/2/8) were incubated with acceptor liposomes (25 µM; POPC/POPE/POPA = 50/40/10) and 20 nM purified Ups1–Mdm35 complex in assay buffer (20 mM Tris-HCl pH 7.5, 150 mM NaCl and 2 mM EDTA) at 25°C. NBD fluorescence was monitored as shown in Fig. 5c. Right panel: Donor liposomes (6.25 µM; POPC/POPE/Egg Liss-Rhod-PE/18:1-12:0 NBD-PS = 50/40/2/8) were incubated with acceptor liposomes (25 µM; POPC/POPE/POPA = 50/40/10) and 20 nM purified Ups1–Mdm35 complex in assay buffer (20 mM Tris-HCl pH 7.5, 150 mM NaCl and 2 mM EDTA) at 25°C. At 0 sec, the protein or buffer was added to the reaction mixture, and NBD fluorescence intensities were set to 0 at 0 sec. Traces show means ± SD of three independent experiments.

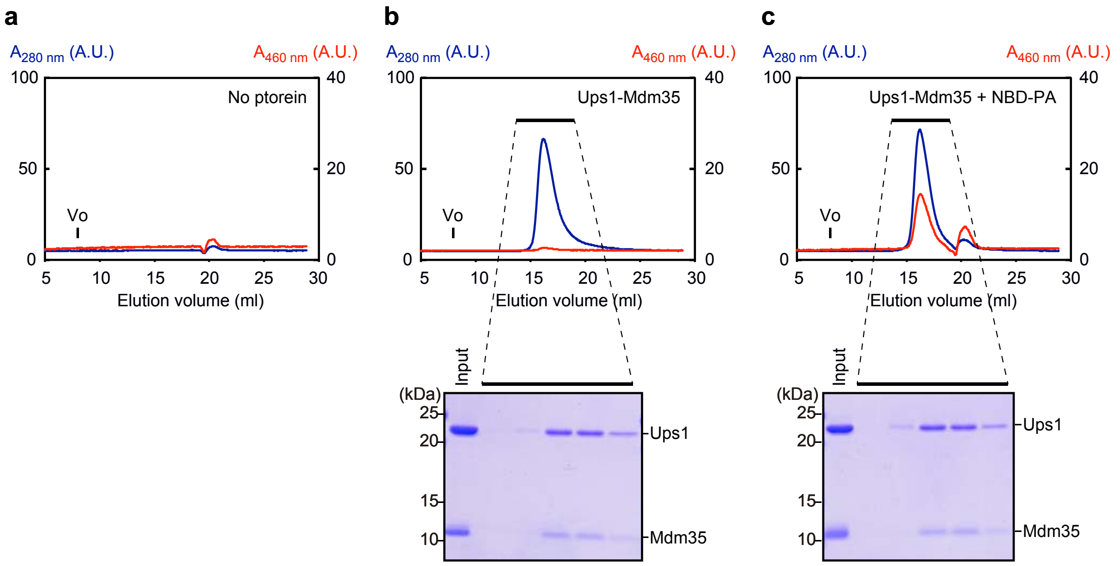

Supplementary Figure 6 | Gel filtration profiles of Ups1–Mdm35 with NBD-PA. Donor liposomes (200 μM; POPC/POPE/18:1-12:0 NBD-PA = 50/40/10) loaded with 12.5% sucrose (**a**), 10 μM Ups1–Mdm35 complex (**b**), and both 10 μM Ups1–Mdm35 complex and donor liposomes (200 μM; POPC/POPE/18:1-12:0 NBD-PA = 50/40/10) loaded with 12.5% sucrose (**c**) were incubated in 20 mM Tris-HCl pH 7.5 and 150 mM NaCl and centrifuged at 150,000 *g* for 1 h. The supernatants separated form the donor liposomes were subjected to gel filtration using a Superdex 200 10/300 GL column. Absorbance at 280 nm and 460 nm indicating the Ups1–Mdm35 complex (blue) and NBD-PA (red), respectively, was monitored. Vo, void volume. Fractions indicated with black bars in the elution profiles (upper) were analyzed by SDS-PAGE and Coomassie staining (lower).

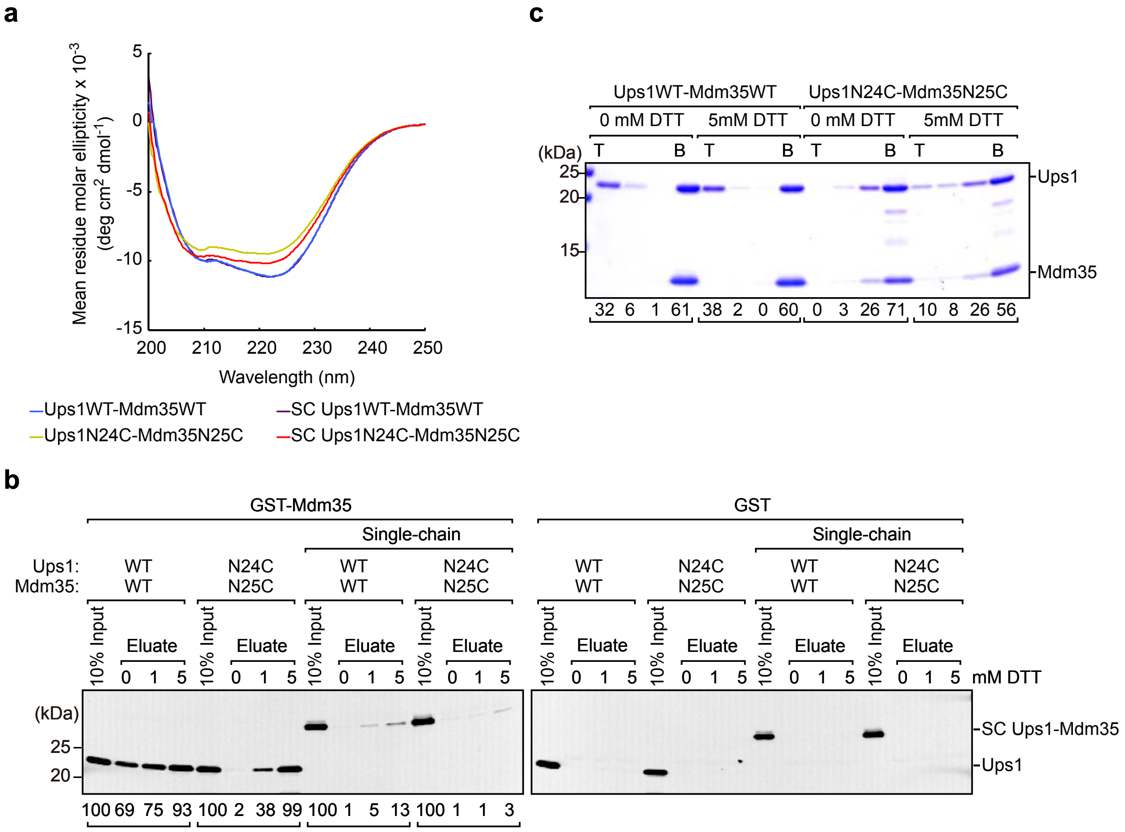

Supplementary Figure 7 | Analyses of the disulfide linked Ups1–Mdm35 mutants. (**a**) CD spectra of Ups1WT–Mdm35WT, Ups1N24C–Mdm35N25C, SC Ups1WT–Mdm35WT and SC Ups1N24C–Mdm35N25C, showing their similar folding. (**b**) 10 μM Ups1WT–Mdm35WT or Ups1N24C–Mdm35N25C, pretreated with the indicated concentrations of DTT for 10 min, were mixed with 10 μM GST or GST-Mdm35, immobilized with 50 μl of GS4B resin, and incubated in 20 mM Tris-HCl pH 7.5 and 150 mM NaCl at 37°C for 15 min. Upon 10-fold dilution of the proteins, proteins bound to the resin were eluted by 10 mM glutathione and analyzed by SDS-PAGE and immunoblotting with the anti-His-tag antibody. (**c**) 5 μM Ups1WT–Mdm35WT or Ups1N24C–Mdm35N25C, pretreated with the indicated concentrations of DTT for 10 min, were incubated with CL-containing liposomes at pH 6.5. Upon 20-fold dilution of the proteins, binding was analyzed as in Fig. 5c. The amounts of Ups1 are shown below the gel (total Ups1 amounts were set to 100).

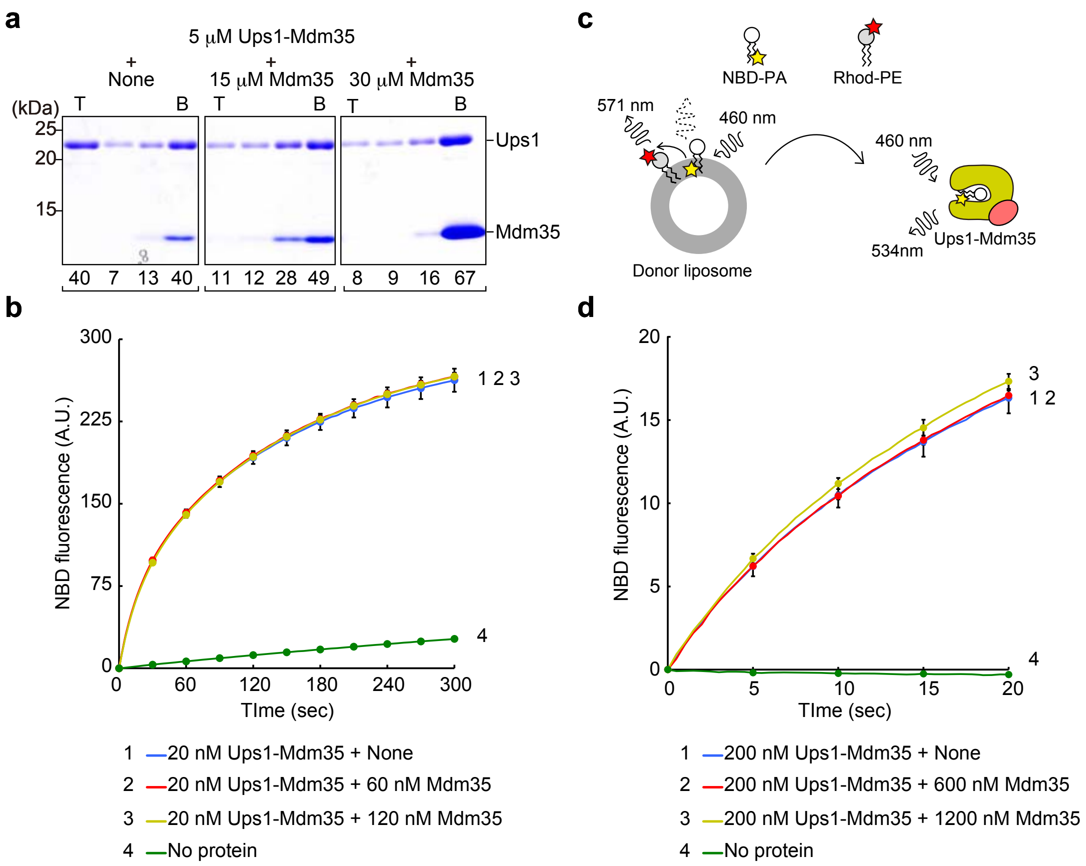

Supplementary Figure 8 | PA transfer and extraction activities of Ups1–Mdm35 in the presence of excess amounts of free Mdm35. (**a**) 5 μM Ups1-Mdm35 was incubated with CL-containing liposomes at pH 6.5 in the presence of the indicated concentrations of Mdm35. Binding was analyzed as in Fig. 5c. The amounts of Ups1 are shown below the gel (total Ups1 amounts were set to 100). (**b**) PA transfer activities of Ups1–Mdm35 in the presence of the indicated concentrations of Mdm35 were analyzed as in Fig. 5a. At 0 sec, the protein or buffer was added to the reaction mixture, and NBD fluorescence intensities were set to 0 at 0 sec. Traces show means ± SD of three independent experiments. (**c**) A schematic diagram of the fluorescent-based PA extraction assay. (**d**) Donor liposomes (6.25 μM; POPC/POPE/Egg Liss-Rhod-PE/18:1-12:0 NBD-PA = 50/40/2/8) were incubated with 200 nM Ups1–Mdm35 in the presence of the indicated concentrations of Mdm35 in 2 ml of assay buffer (20 mM Tris-HCl pH 7.5, 150 mM NaCl and 2 mM EDTA) at 25°C. At 0 sec, the protein or buffer was added to the reaction mixture, and NBD fluorescence intensities were set to 0 at 0 sec. Traces show means ± SD of three independent experiments.

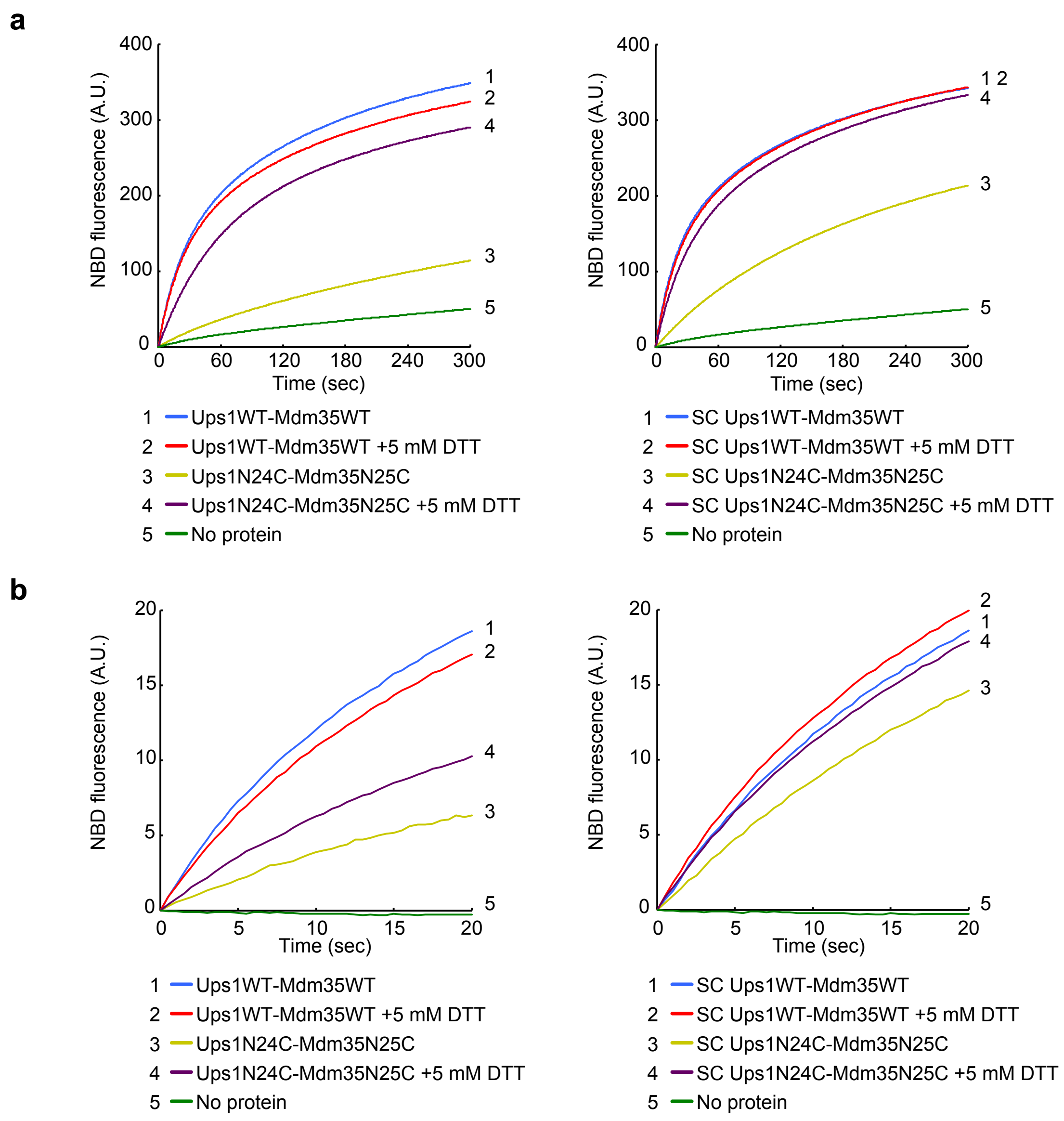

Supplementary Figure 9 | PA transfer and extraction activities of the disulfide linked Ups1–Mdm35 complex. (**a**) PA transfer activities of Ups1WT–Mdm35WT and Ups1N24C–Mdm35N25C (left) or single-chain (SC) Ups1WT–Mdm35WT and SC Ups1N24C–Mdm35N25C (right), pretreated with 5 mM DTT for 10 min, were analyzed as in Fig. 5a. At 0 sec, the protein or buffer was added to the reaction mixture with 500-fold dilution of the protein, and NBD fluorescence intensities were set to 0 at 0 sec. We confirmed that 10 μM DTT (final concentration in the reaction mixture) does not affect the NBD fluorescence. (**b**) PA extraction activities of Ups1WT-Mdm35WT and Ups1N24C–Mdm35N25C (left) or single-chain (SC) Ups1WT–Mdm35WT and

SC Ups1N24C–Mdm35N25C (right), pretreated with 5 mM DTT, were analyzed as in Supplementary Fig. 8d. At 0 sec, the protein or buffer was added to the reaction mixture with 500-fold dilution of the protein, and NBD fluorescence intensities were set to 0 at 0 sec.

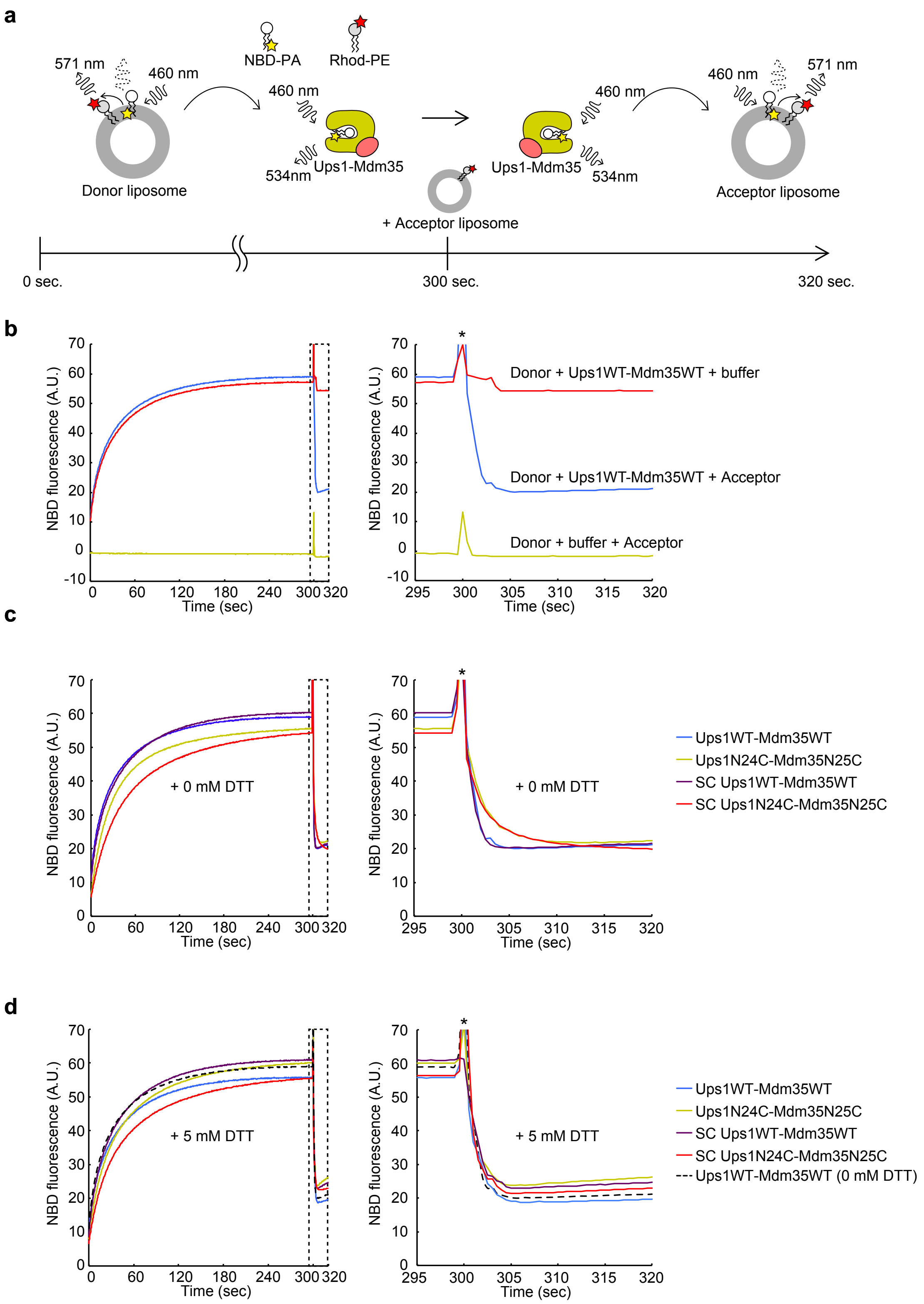

Supplementary Figure 10 | PA release assay. (**a**) A schematic diagram of the fluorescent-based PA release assay (see details in Methods). (**b**) Donor liposomes (6.25 μM; POPC/POPE/Egg Liss-Rhod-PE/18:1-12:0 NBD-PA = 50/40/2/8) were incubated with or without 200 nM purified Ups1–Mdm35 in 2 ml

of assay buffer (20 mM Tris-HCl pH 7.5, 150 mM NaCl and 2 mM EDTA) at 25°C for 300 sec., then acceptor liposomes (25 μM; POPC/POPE/Egg Liss-Rhod-PE/POPA = 50/40/2/8) or assay buffer were added, and the NBD fluorescence was monitored. A magnified view of the data in the broken box in the left panel is shown in the right panel. Asterisk marks addition of acceptor liposomes. (**c**), (**d**) PA release activities of Ups1–Mdm35 mutants. Ups1WT–Mdm35WT, Ups1N24C–Mdm35N25C, SC Ups1WT–Mdm35WT or SC Ups1N24C–Mdm35N25C pretreated without (**c**) or with (**d**) 5 mM of DTT were, upon 500-fold dilution of the protein, analyzed as in (b). A magnified view of the data in the broken box in the left panel is shown in the right panel. Asterisk marks addition of acceptor liposomes.

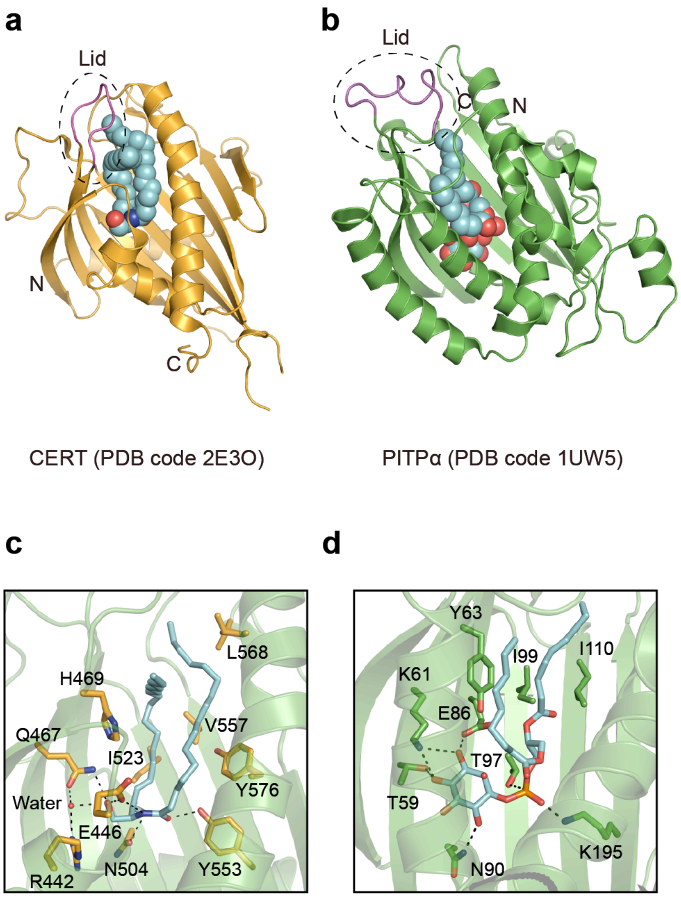

Supplementary Figure 11 | Structures of the START domains. Ribbon diagrams of the crystal structures of CERT START domain in a complex with C16-ceramide (PDB code 2E3O) (**a**) and PITPα in a complex with phosphatidylinositol (PDB code 1UW5) (**b**). The lid regions are colored in magenta and the bound ligands are shown in space-filling form with C cyan, O red, and P orange. Magnified views showing the detailed interaction around the ceramide molecule (**c**) and phosphatidylinositol molecule (**d**). Residues and a water molecule responsible for ligand recognition are shown as stick models and a sphere model, respectively. Broken lines designate possible hydrogen bonds.

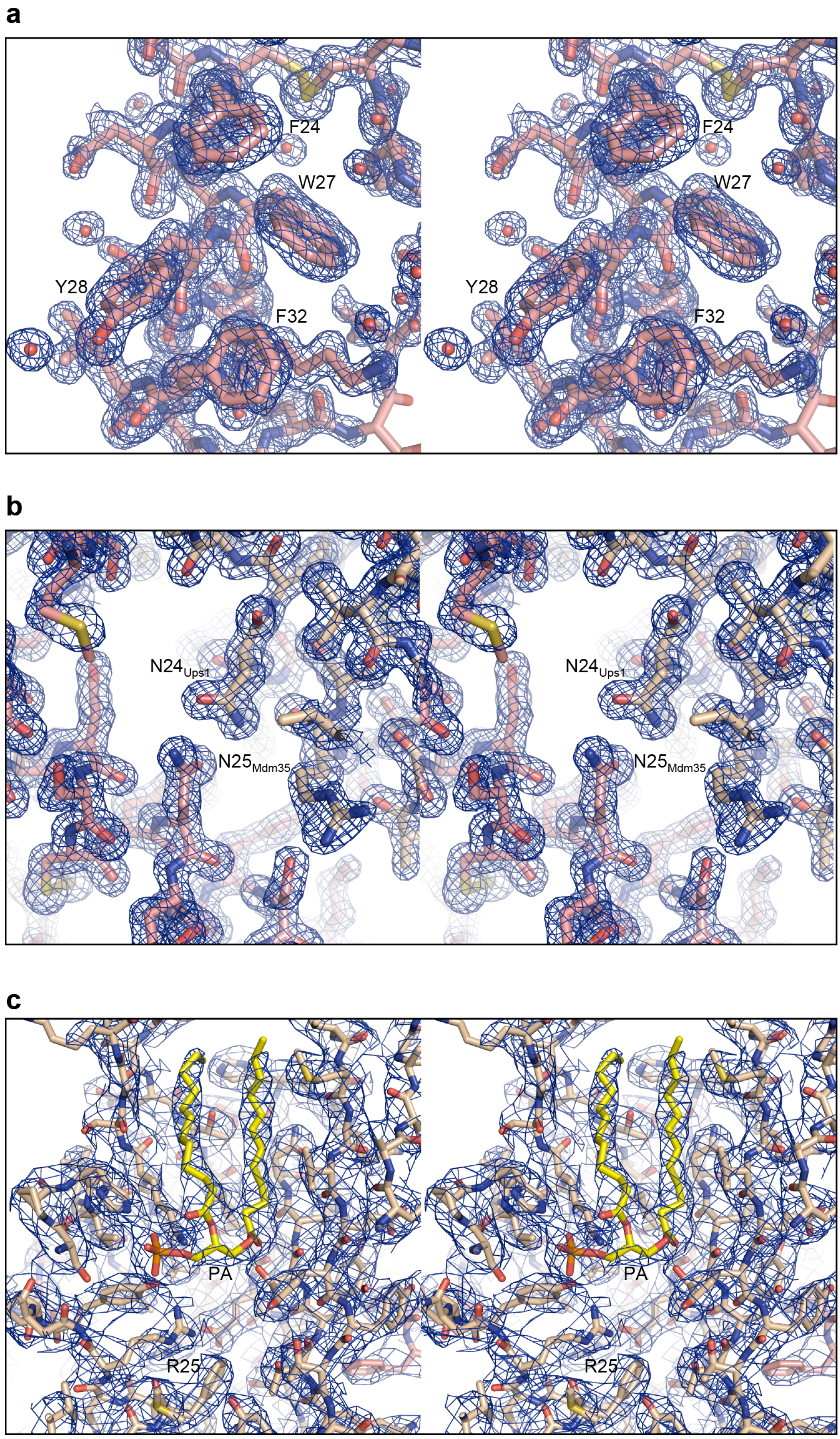

Supplementary Figure 12 | Examples of electron density. Stereo views of the 2F_o-F_c electron density maps of free Mdm35 (**a**), the Ups1–Mdm35 complex (**b**) and the Ups1–Mdm35–PA complex (**c**), countered at 1.2 σ level. The final models are shown in stick model; C atoms of Mdm35, Ups1 and a PA molecule are colored in pink, light brown, and yellow, respectively.

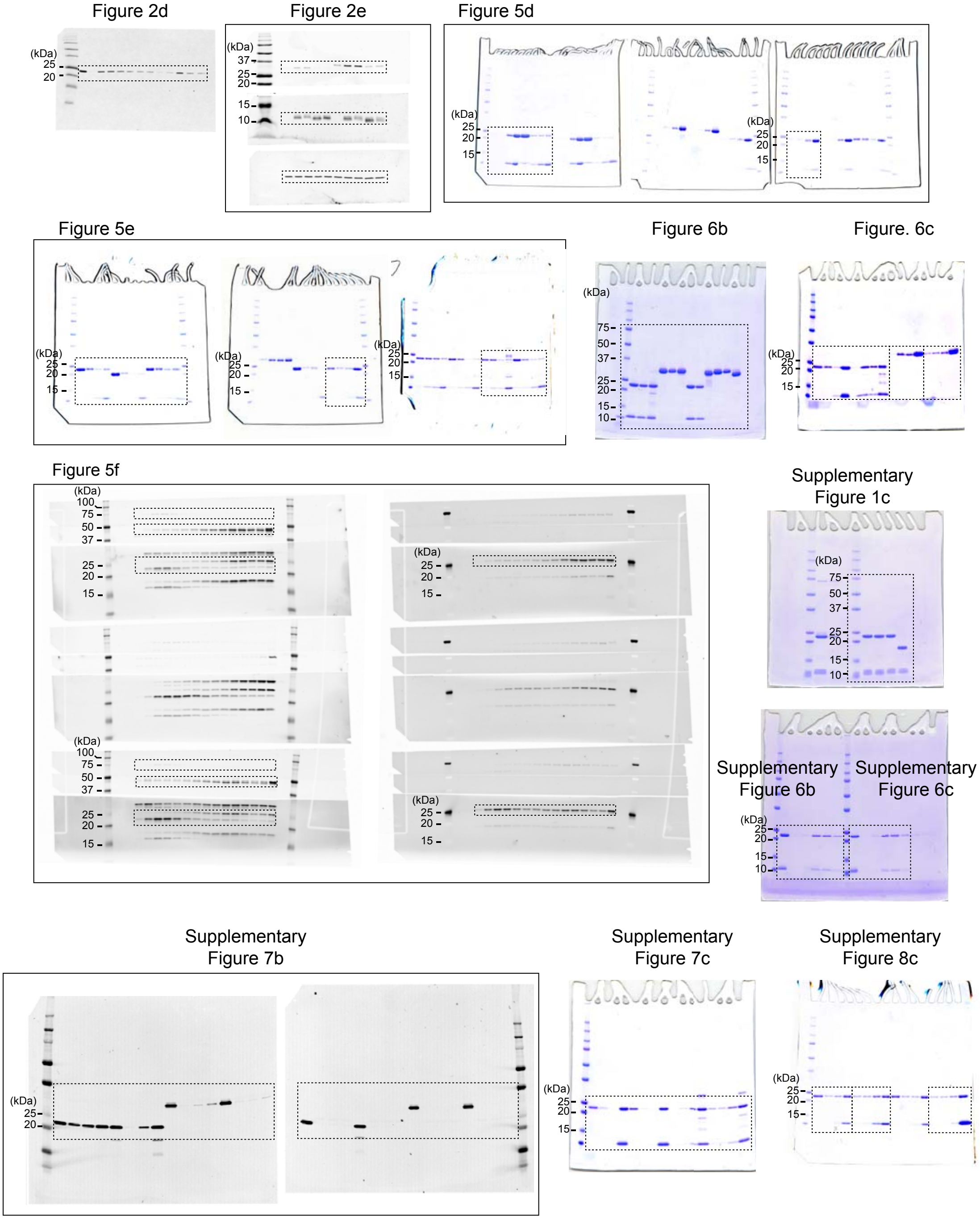

Supplementary Figure 13 | Uncropped images for immunoblotting and SDS-PAGE with CBB staining. Broken boxes mark the borders of the final cropped images.
